# Talin1 dysfunction is genetically linked to systemic capillary leak syndrome

**DOI:** 10.1172/jci.insight.173664

**Published:** 2024-12-20

**Authors:** Naama Elefant, Georgia Rouni, Christina Arapatzi, Danit Oz-Levi, Racheli Sion-Sarid, William J.S. Edwards, Neil J. Ball, Shira Yanovsky-Dagan, Alana R. Cowell, Vardiella Meiner, Vladimir Vainstein, Sofia Grammenoudi, Doron Lancet, Benjamin T. Goult, Tamar Harel, Vassiliki Kostourou

**Affiliations:** 1 Department of Genetics, Hadassah Medical Organization, Jerusalem, Israel.; 2 Institute of BioInnovation, Biomedical Sciences Research Centre “Alexander Fleming,” Vari-Athens, Greece.; 3 Department of Biology, University of Patras, Patras, Greece.; 4 Molecular Genetics, Weizmann Institute of Science, Rehovot, Israel.; 5 Pediatric Intensive Care Unit, Wolfson Medical Center, Holon, Israel.; 6 School of Biosciences, University of Kent, Canterbury, Kent, United Kingdom.; 7 Faculty of Medicine, Hebrew University of Jerusalem, Jerusalem, Israel.; 8 Department of Hematology, Hadassah Medical Organization, Jerusalem, Israel.; 9 Department of Biochemistry, Cell & Systems Biology, Institute of Systems, Molecular & Integrative Biology, University of Liverpool, Liverpool, United Kingdom.

**Keywords:** Cell biology, Vascular biology, Cell migration/adhesion, Endothelial cells, Integrins

## Abstract

Systemic capillary leak syndrome (SCLS) is a rare life-threatening disorder due to profound vascular leak. The trigger and the cause of the disease are currently unknown and there is no specific treatment. Here, we identified a rare heterozygous splice-site variant in the *TLN1* gene in a familial SCLS case, suggestive of autosomal dominant inheritance with incomplete penetrance. Talin1 has a key role in cell adhesion by activating and linking integrins to the actin cytoskeleton. This variant causes in-frame skipping of exon 54 and is predicted to affect talin’s C-terminal actin-binding site (ABS3). Modeling the SCLS-*TLN1* variant in *TLN1*-heterozygous endothelial cells (ECs) disturbed the endothelial barrier function. Similarly, mimicking the predicted actin-binding disruption in *TLN1*-heterozygous ECs resulted in disorganized endothelial adherens junctions. Mechanistically, we established that the SCLS-*TLN1* variant, through the disruption of talin’s ABS3, sequestrates talin’s interacting partner, vinculin, at cell–extracellular matrix adhesions, leading to destabilization of the endothelial barrier. We propose that pathogenic variants in *TLN1* underlie SCLS, providing insight into the molecular mechanism of the disease that can be explored for future therapeutic interventions.

## Abstract 2

Systemic capillary leak syndrome (SCLS) is a rare potentially lethal disease with unknown etiology and non-specific treatment. Here, we established a heterozygous splice variant of talin1, a key cell adhesion protein, as a genetic link to a familial SCLS

## Graphical abstract

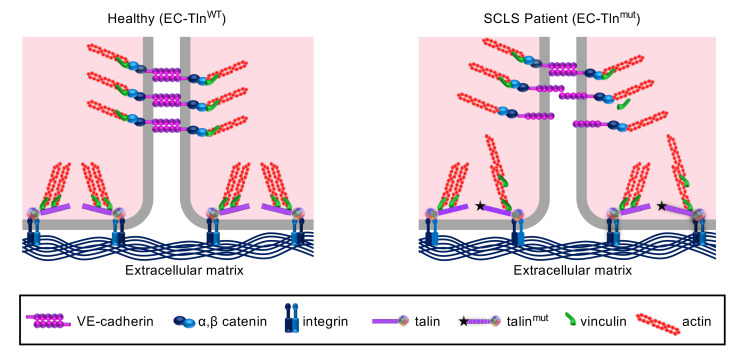

## Introduction

Systemic capillary leak syndrome (SCLS) is a rare and life-threatening condition that was first described in 1960 by Clarkson and is also referred to as Clarkson disease (1). It is characterized by recurrent, transient, and spontaneous episodes of massive shifts of intravascular fluids into peripheral tissues and presents with hypovolemic shock, hemoconcentration, and hypoproteinemia (2). Only a few hundred sporadic cases are described world-wide, with presenting ages spanning from early childhood to adulthood. Frequency and severity of episodes vary between patients. Episodes are frequently preceded by viral infections and about half of the patients report preceding symptoms, including oligo-anuria, fatigue, edema, syncopal episodes, abdominal pain, nausea, myalgias, polydipsia, and a sudden increase in body weight (3, 4). Episodes are usually treated in the setting of an emergency department or an intensive care unit, with massive infusions of intravenous saline. There are no established acute pharmacologic therapies for SCLS. In several reports, the use of intravenous immunoglobulins, terbutaline combined with aminophylline, infliximab, or anti-VEGF antibodies are described with varying degrees of success (4, 5). Treatment is challenging, since the provided fluids are promptly “third-spaced” due to the vascular leakage, leading to anasarca (with relative sparing of the lungs). As a result, compartment syndrome may develop in the peripheral limbs, frequently necessitating fasciotomies. Symptoms typically resolve spontaneously after 48–96 hours without specific treatment other than intense hemodynamic support and treatment of complications.

The etiology of SCLS remains unknown, with most current hypotheses presupposing exaggerated or abnormal cellular signaling, either in vascular endothelium or inflammatory cells (6, 7). Up to 80% of adult patients with SCLS are found to have a monoclonal gammopathy of unknown significance, but the role of this finding in the pathogenicity of the disease has not been established and is not considered essential for the diagnosis of SCLS (5). In an attempt to discover germline mutations underlying the disease, several papers have conducted sequencing studies of patients with SCLS. In one publication, genome-wide single nucleotide polymorphism (SNP) analysis was conducted for 12 patients, revealing several loci and SNPs associated with the disease (8). In a more recent study, exome sequencing was performed for 16 adult and pediatric patients with SCLS, and several candidate genes were proposed, but no uniform germline exonic variants were found (9).

Here, we report on the results of the genetic investigation of 3 SCLS patients and segregation analysis from an extended family pedigree suggestive of autosomal dominant inheritance with incomplete penetrance. We identified a heterozygous splice-site variant, c.7188+2T>C, in the *TLN1* gene affecting the C-terminal region of the talin1 protein. Talin1 is a key component of integrin-mediated cell–extracellular matrix (cell-ECM) adhesions and has important functions in integrin activation, mechanotransduction, cell attachment, and spreading (10, 11). Talin1 is a 2541 amino acid protein that comprises an N-terminal head domain that binds integrins connected to a C-terminal rod region comprising 13 α-helix bundles, R1–R13, culminating in a dimerization domain (DD) helix (12, 13). The talin rod region contains 11 vinculin-binding sites (VBSs) and 2 actin-binding sites (ABSs) that can be conformationally regulated by application of cytoskeletal forces. Several studies have demonstrated a significant role for talin1 in physiological organ function and cancer (14, 15). In the vascular system, talin1 is essential for embryonic and postnatal blood vessel development and tumor angiogenesis (16–18). Recently, it was shown that endothelial cell–specific (EC-specific) deletion of talin1 caused intestinal microvascular bleeding because of defective integrin activation (19). In addition, studies have shown that *TLN1* was downregulated in aortic tissue of patients with aortic dissection, and rare heterozygous missense variants in *TLN1* have been found in individuals with familial spontaneous coronary artery dissection (20, 21). Together, these studies highlight a critical role for talin1 in vascular endothelium.

Here, we report a *TLN1* variant found in an extended pedigree with SCLS and establish that this heterozygous SCLS-*TLN1* mutation disrupts the integrity of the endothelial barrier. We show that dysfunction in the C-terminal ABS (ABS3) of talin results in disorganized endothelial adherens junctions (AJs) similarly to the SCLS-*TLN1* mutant. Mechanistically, we propose that the SCLS-*TLN1* variant interferes with the ability of talin to bind actin, which perturbs the localization of talin’s partner vinculin to AJs. This results in defective remodeling of cell-cell junctions and loss of vascular integrity. Our data indicate a genetic germline etiology and provide a mechanistic insight into the cause of SCLS that can be exploited for both diagnostic and therapeutic interventions.

## Results

### Clinical reports.

**Figure 1:**
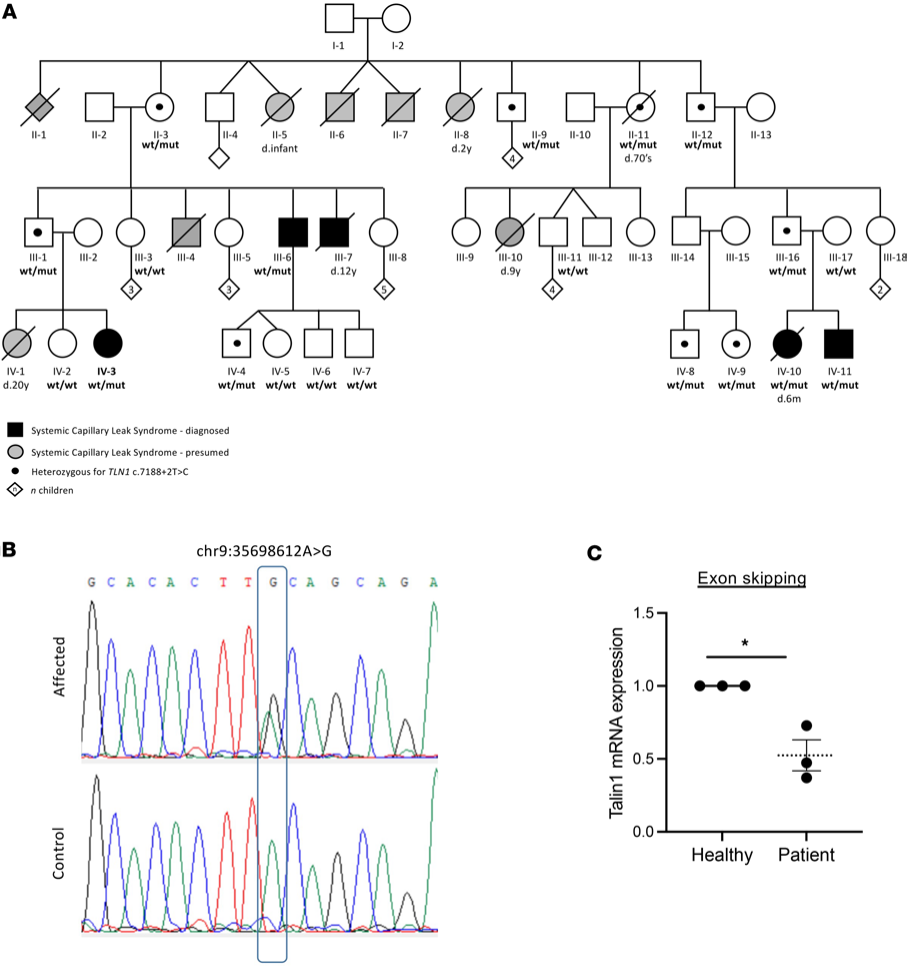
Pedigree of the familial case of SCLS and identification of an SCLS-*TLN1* variant. (**A**) Pedigree indicating affected individuals as black-filled shapes. Gray-filled shapes indicate individuals suspected to have been affected, where DNA was not available for testing. Diagonal black line indicates deceased (d. time of death). Black dots indicate nonsymptomatic individuals heterozygous for the SCLS variant. Numbers in diamond shapes indicate number of children. Inheritance was presumed to be autosomal dominant with incomplete penetrance. (**B**) Sanger sequencing at the DNA level, from 3 living patients (IV-3, III-6, and IV-11) showing heterozygous missense variant of splice site at exon 54 (c.7188+2T>C). (**C**) Quantitative real-time PCR analysis showing the fold difference in the mRNA expression of exon 54 between fibroblasts derived from the patient (IV-11) and the healthy relevant (III-17) normalized to the *RPLP1* reference gene. Data shown are mean ± SEM, from 3 independent experiments. *P = 0.011 relative to WT values by unpaired, 2-tailed *t* test.

The proband (Figure 1A, Individual IV-3) presented with her first episode of SCLS to the ER with severe hypotension, hypovolemia, and hemoconcentration and was stabilized and treated with intravenous fluids. A proband’s relative (Individual III-6) reported episodes of SCLS mostly in his childhood and teenage years. Another proband’s relative (Individual IV-11) suffered episodes of SCLS from 6 months of age. In early years, he suffered a severe attack with cerebral edema, which upon resolution required rehabilitation for several months. In childhood and adolescence, he suffered monthly episodes and currently experiences an episode once every 4–6 months. Another relative (Individual IV-10) died at childhood. Archived medical records of another relative (Individual III-7) describing episodes of abdominal pain, hypotension, hemoconcentration, and loss of consciousness that required treatment with intravenous fluids, are suggestive of SCLS. In addition, several members of the extended family died at a young age, possibly having SCLS without being properly diagnosed. Detailed clinical reports are available upon request.

### Identification of a heterozygous variant in TLN1.

Exome sequencing was undertaken on the 3 living patients with SCLS (IV-3, III-6, and IV-11), in search of an underlying genetic diagnosis. Following alignment to the reference genome (hg19) and variant calling, variants were filtered out if they were off target (>8 bp from splice junction), synonymous, or had a minor allele frequency of greater than 0.01 in the gnomAD database. Due to the presumed autosomal dominant mode of inheritance with incomplete penetrance, the analysis targeted heterozygous rare variants that were shared among the affected individuals. Only 2 variants survived this filtering (Supplemental Table 1; supplemental material available online with this article; https://doi.org/10.1172/jci.insight.173664DS1). All individuals shared a splice-site variant located in intron 54 of the *TLN1* gene (chr9: g.35698612A>G [hg19]; NM_006289.4: c.7188+2T>C) (Figure 1B). This splice-site variant was not found in gnomAD or in our in-house database of approximately 13,000 exomes, and was predicted to alter splicing (SpliceAI) (22). Following this finding, segregation analysis was then performed on several members of the extended family. All affected individuals and obligatory carriers were heterozygous for the variant. Consistent with the incomplete penetrance, several non-manifesting carriers were identified (Figure 1A, ages 13–75 years). These individuals had no history of syncope or sudden hypotension. Taken together, our genetic analysis identified a heterozygous splice variant with incomplete penetrance and variable expressivity in this familial case of SCLS.

### The SCLS-TLN1 variant causes exon skipping at the RNA level.

To determine whether the SCLS-*TLN1* variant affects splicing, we analyzed the cDNA sequence of 2 patients. Two tissues were analyzed (lymphocytes and fibroblasts) to examine whether the effect of the variant was tissue specific. Reverse transcription PCR on RNA extracted from both tissues revealed the existence of a lower molecular weight band in the affected individual in addition to the wild-type (WT) band, suggesting exon skipping (Supplemental Figure 1A). Sequencing of the lower band after gel extraction revealed skipping of exon 54 (Supplemental Figure 1B). Thus, we concluded that the *TLN1* c.7188+2T>C variant led to a heterozygous in-frame deletion of 63 nucleotides. To quantify the percentage of talin1 transcripts with the exon skipping event, real-time PCR was performed on cDNA from fibroblasts derived from an SCLS patient (IV-11) and a healthy control relative (III-17) using specific primers that targeted the affected area. The patient fibroblasts displayed a mean 50% reduction in the mRNA transcript that contains exon 54, confirming the mis-splicing effect of the identified SCLS-*TLN1* variant (Figure 1C).

### The SCLS-TLN1 variant results in disturbance of the 13th talin rod domain, R13.

**Figure 2:**
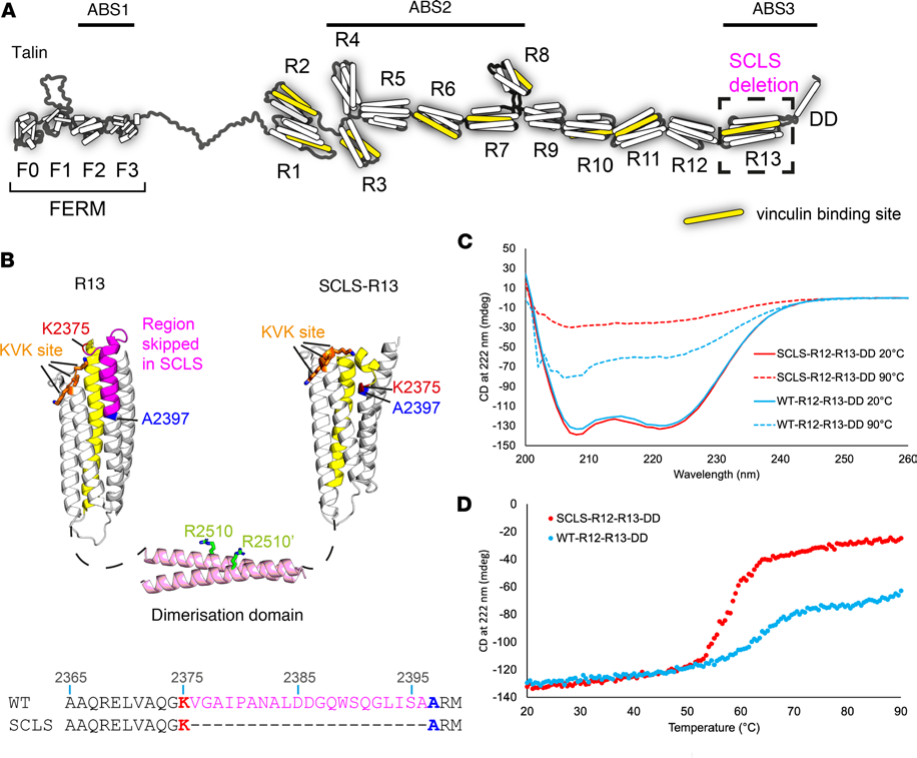
Structural analysis of the SCLS-*TLN1* variant protein indicates a destabilized R13 domain. (**A**) The domain structure of talin contains an N-terminal FERM domain and a large rod region consisting of 13 helical bundles, R1–R13, ending in a dimerization domain (DD). The 3 actin-binding sites (ABSs) are highlighted. The 11 vinculin-binding sites (VBSs) are shown in yellow. The R13 domain where the SCLS variant leads to deletion of 21 aa is highlighted. (**B**) The structure of WT R13 (left) and the predicted structural model of SCLS-R13 (right) connected by the DD. Sequence alignment of the WT (top) and SCLS (bottom) region of R13 containing the skipped exon (bottom). The KVK site (residues K2443/V2444/K2445) and the R2510 site that are required for actin binding are shown in orange and green, respectively. The 21 residues skipped in SCLS are shown in magenta. K2375 (red) and A2397 (blue) are the residues immediately before and after the skipped region. To highlight the distortion, the VBS helix at the back of the WT protein is shown in yellow; in SCLS, this helix is broken. (**C** and **D**) Circular dichroism (CD) analysis. (**C**) Far-UV spectral analysis (between 200 and 260 nm wavelengths) of 0.5 mg/mL WT-R12-R13-DD (blue) and SCLS-R12-R13-DD (red) at 20°C and at 90°C. (**D**) Melting curves at 222 nm for 0.5 mg/mL WT-R12-R13-DD (blue) and SCLS-R12-R13-DD (red), measuring thermal stability over increasing temperature from 20°C to 90°C.

The deletion of exon 54 results in the in-frame deletion of 21 amino acids from the talin primary sequence. These residues, V2376–A2396 inclusive, are situated toward the C-terminus of the talin protein in the 13th rod domain, R13 (Figure 2, A and B). The structure of R13 is a 5-helix bundle and the SCLS-*TLN1* variant results in deletion of part of the 59th helix of talin1 (Figure 2B, magenta). R13 is an important domain for talin function, as it forms part of ABS3 in conjunction with the adjacent DD (Figure 2B) (12). Binding of actin to ABS3 has been mapped to 2 interfaces on the R13-DD module, one on R13 centered around a basic amino acid (KVK) surface (Figure 2B, orange), and a second on the DD coiled coil centered around R2510 (Figure 2B, green). Mutations that disrupt either the KVK (K2443D/V2444D/K2445D) or R2510 (R2510A) surfaces have a significant impact on actin binding to ABS3 without perturbing structural integrity or dimerization (12). Helix 58 of R13 (yellow) is also a VBS. Because of the proximity of the SCLS deletion to the ABSs and VBSs in R13, we wanted to establish the impact of the SCLS deletion on the biochemical and biophysical properties of talin.

To visualize the effects of the exon skipping on the structural integrity of R13, we first generated a structural model of SCLS-R13 using the protein prediction software AlphaFold2 (23). Comparison of the structure of WT-R13 (Figure 2B) with the structural model of SCLS-R13 (Figure 2B) revealed that the SCLS deletion severely distorts the R13 structure and introduces a kink into the region of R13 known to be important for actin binding.

We next generated expression constructs of the ABS3 domain (R13-DD) and the R12-R13-DD, both WT and with the SCLS deletion and attempted to express and purify them from *E*. *coli* using standard protocols. As previously, the WT R13-DD and R12-R13-DD expressed and purified nicely; however, the SCLS-R13-DD was insoluble, precluding it from further study. The SCLS-R12-R13-DD behaved slightly better and was able to be studied. Far-UV circular dichroism (CD) analysis supported the modeling, as both WT-R12-R13-DD and SCLS-R12-R13-DD had very similar CD spectra at 20°C, with the characteristic double minima peaks at 208 and 222 nm indicative of both being predominantly α-helical (Figure 2C). R13 is the most stable domain in talin unfolding at greater than 90°C and this stability is evident in the thermal melting curves of the WT-R12-R13-DD where 2 unfolding events are evident, one at approximately 60°C, which is the R12 unfolding, and then the start of a second unfolding event at 90°C (Figure 2D). Even at 90°C, WT R13 was still mostly folded, which is also seen in the CD spectra (Figure 2C). In contrast, the SCLS-R12-R13-DD showed a single unfolding event at 59°C, after which the whole protein was fully unfolded (Figure 2D). This biophysical analysis confirms that the SCLS deletion destabilizes the R13 domain.

We next wanted to study the binding of the WT-R12-R13-DD and SCLS-R12-R13-DD constructs to actin using the well-established actin cosedimentation assay. However, while WT-R12-R13-DD remained entirely in the supernatant when centrifuged at high speeds, SCLS-R12-R13-DD pellets by itself (Supplemental Figure 2A), indicating that the deletion renders the protein only sparingly soluble and aggregation prone and is not suitable for cosedimentation-based assays.

Finally, we assessed the ability of SCLS-R12-R13-DD to bind vinculin. Whereas actin binding to talin involves a surface of the folded domains engaging the actin filament, vinculin binding requires the talin rod domain to unfold and expose the VBS helix to enable binding (24). Given that the SCLS deletion destabilizes R13, we examined how this affected vinculin binding to R13 by performing size exclusion chromatography (SEC) using talin and the talin-binding domain 1 of vinculin (Vd1). Despite being 21 amino acids smaller than the WT, SCLS-R12-R13-DD eluted slightly earlier, suggesting that it is less compact in structure (Supplemental Figure 2B). The SEC experiment in the presence of a 1:1 ratio of talin and vinculin Vd1 domain showed that both WT-R12-R13-DD and SCLS-R12-R13-DD bound equally well to Vd1 at 25°C (Supplemental Figure 2B).

In summary, the result of the exon skipping at the protein level substantially disrupted the talin R13 domain, which impacted its stability and likely its ability to bind actin. However, vinculin binding to R13 was not noticeably affected.

### The SCLS-TLN1 variant does not affect talin localization at cell-ECM adhesions or patient fibroblast adhesion properties.

Mutations at splice sites often lead to decreased protein expression (25). To examine this, we performed Western blotting and immunofluorescence analyses in skin fibroblasts derived from patient IV-11 and an unaffected healthy relative control (III-17). We could not detect any change in talin1 protein expression or localization at adhesion sites in patient and control fibroblasts (Supplemental Figure 3, A and C). Since fibroblasts also express the closely related *TLN2* gene (26), we investigated whether the expression of talin2 protein is altered in compensation to the SCLS-*TLN1* variant. Similar to talin1, talin2 expression and localization at adhesion sites was unaffected in patient and control fibroblasts (Supplemental Figure 3, B and C).

As talin is a critical regulator of cell-ECM adhesions (16, 27), we investigated the effect of the SCLS-*TLN1* variant on cell-ECM adhesion formation and signaling. Immunofluorescence and Western blot analysis in patient and control fibroblasts revealed that the SCLS-*TLN1* variant did not change the expression or the localization of key adhesion proteins, including paxillin and vinculin, and did not affect the number or the size of cell-ECM adhesions (Supplemental Figure 4, A and B). Additional analysis of cell-ECM adhesion signaling revealed similar activation of p-Y397-FAK and p-Y31-paxillin, in patient and control skin fibroblasts (Supplemental Figure 4, C and D). In accordance with these findings, activation of major downstream signaling pathways, including p-Y44/42-MAPK and p-Akt, were not affected (Supplemental Figure 5, A and B).

Given the established role of talin1 in integrin activation (10), we examined whether the SCLS-*TLN1* variant alters integrin activation in patient and control fibroblasts. Immunofluorescent staining and flow cytometric analysis using an activation-epitope-reporting antibody against the integrin β1 subunit showed that both basal and Mn^2+^-induced activation of integrin β1 was preserved in patient fibroblasts (Supplemental Figure 4, E and F). In addition, total levels of integrin β1 expression (Supplemental Figure 4E) and surface availability, as determined by flow cytometry (Supplemental Figure 4G), did not change significantly in patient and control fibroblasts. Taken together, these data indicate that the SCLS-*TLN1* variant does not affect integrin activation.

To study the functionality of cell-ECM adhesions, we performed live-cell imaging and confirmed that the SCLS-*TLN1* variant does not impair cell adhesion or spreading on different substrates, including plastic, collagen, and fibronectin (Supplemental Figure 6, A–C, and Supplemental Video 1).

### Platelet function is unaffected in an SCLS patient.

Previous studies have shown that disruption of *TLN1* in platelets causes defects in platelet aggregation, thrombus formation, and extensive bleeding in mice (28, 29). Specifically, talin-induced integrin activation has been shown to be required for fibrin clotting (30). Platelet dysfunction can lead to prolonged bleeding, which might contribute to SCLS symptoms. To examine whether the SCLS-*TLN1* variant affects the patient’s normal platelet activity, we performed ex vivo platelet adhesion studies. Platelets derived from both an SCLS patient and an unaffected relative donor had normal size and displayed normal aggregation at the surface (Supplemental Figure 7). Additionally, none of the patients showed any signs of aberrant clotting in their medical history or clinical examination during an attack. Together, these observations suggest that platelet function is not overly affected by the SCLS-*TLN1* variant.

### The SCLS-TLN1 mutant disrupts the integrity of endothelial AJs.

Given the normal adhesion morphology and function of patient fibroblasts and the characteristic vascular leakiness associated with the disease, we hypothesized that the ECs that form blood vessels are the key cellular player in SCLS. Thus, we set out to model the effect of the SCLS-*TLN1* variant in primary ECs. We have shown previously that ECs express only talin1, and that talin2 is not expressed upon deletion of talin1 as a compensatory mechanism (16). To model the haploinsufficiency of the SCLS-*TLN1* mutation found in the patients, we used mouse primary ECs isolated from an inducible EC-specific talin1 mouse model (*pdgfBB*iCreER^T2^ *TLN1^fl/+^*). We transfected these ECs with GFP-talin1 expression constructs lacking the 21 amino acids of exon 54 (EC-Tln^Δex54^) or WT full-length protein (EC-Tln^WT^). Tamoxifen-induced Cre activation in vitro results in genetic disruption of only one *TLN1* allele and thus ensures expression of the SCLS talin1 protein or control WT talin1, in a talin1-heterozygous background. Both constructs were fused to GFP to enable visualization of the expressed proteins. We verified that both the SCLS-*TLN1* mutant and the control talin1 proteins were expressed and localized at cell-ECM adhesions (Supplemental Figure 8).

**Figure 3:**
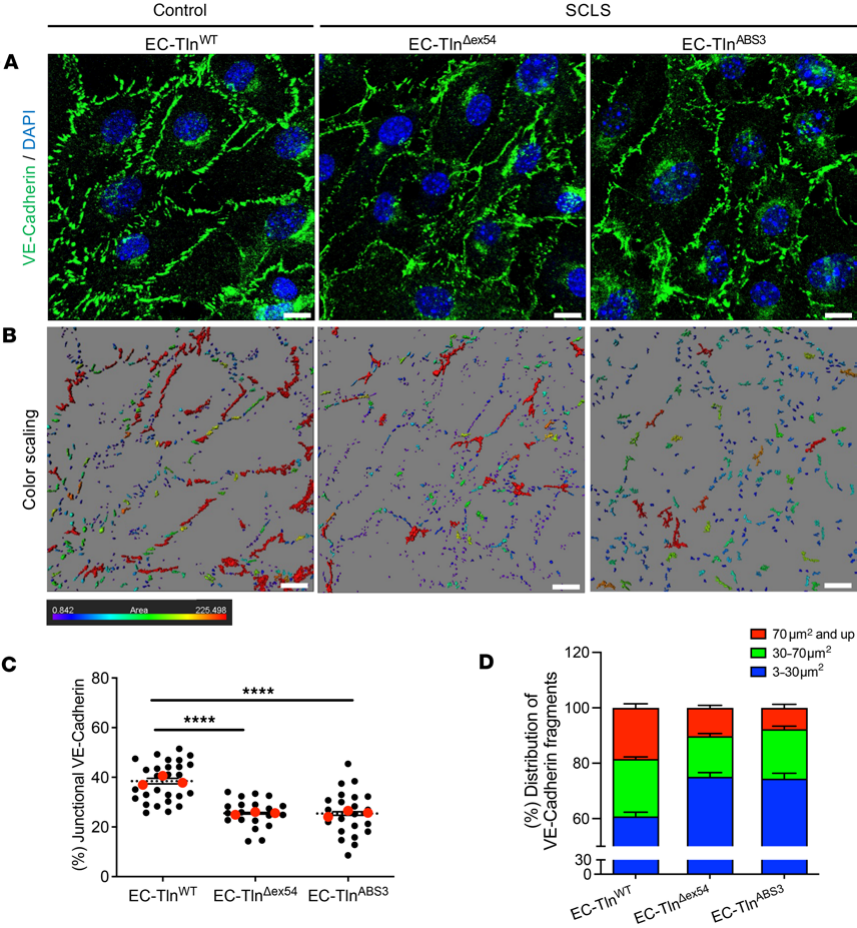
Disruption of cell-cell junctions in SCLS-*TLN1* mutant endothelial monolayers. (**A**) Representative confocal 3D images of VE-cadherin (green) immunostained confluent monolayers of heterozygous-talin1 primary ECs transfected with full-length talin1 protein (EC-Tln^WT^) or SCLS-*TLN1* mutant lacking the 21 aa of exon 54 (EC-Tln^Δex54^) or the talin1 ABS3 mutation, R2510A (EC-Tln^ABS3^). Nuclei were stained with DAPI (blue). (**B**) Color scaling of VE-cadherin, whereby the red color is the highest continuous staining area (>70 μm^2^), decreasing to smaller areas marked by different colors until it reaches the lowest measurements, which are of blue-violet color (<30 μm^2^), analyzed by IMARIS. Scale bars: 10 μm. (**C**) Graph displays the quantification of the continuous junctional area, as represented by the percentage of staining surfaces greater than 30 μm^2^ versus the total staining area. Data represent the mean area per field of monolayer. *n* fields of view analyzed: EC-Tln^WT^ = 27; EC-Tln^Δex54^ = 21; EC-Tln^ABS3^ = 22. Red dots represent the mean ± SEM of 3 independent experiment. ****P < 0.0001 by 1-way ANOVA with Dunnett’s multiple-comparison test. (**D**) Graph displays the distribution of 3 different indexes of junctional fragments as a percentage of the total staining surfaces. Data represent the mean ± SEM of 3 independent experiments. *n* of field of views analyzed: EC-Tln^WT^ = 25; EC-Tln^Δex54^ = 22; EC-Tln^ABS3^ = 22.

Using the SCLS-*TLN1* mutant ECs, we next set out to characterize the EC-EC junctions that we visualized using immunofluorescent staining of VE-cadherin, a key component and regulator of AJs (31). Morphological examination of EC confluent monolayers formed by WT ECs or heterozygous control ECs expressing full-length talin1 protein (EC-Tln^WT^) displayed continuous and well-formed AJs (Figure 3A and Supplemental Figure 9). However, SCLS-*TLN1* mutant ECs (EC-Tln^Δex54^) revealed severely disrupted AJs compared with the control EC-Tln^WT^ (Figure 3A). The fragmentation of AJs caused by the SCLS mutation is more clearly visualized using a color scaling, where red indicates large and continuous VE-cadherin areas (>70 μm^2^) and blue-violet highlights small and unstable AJs (<30 μm^2^) (Figure 3B). Quantification of VE-cadherin staining showed decreased localization of VE-cadherin at cell-cell borders (Figure 3C) and discontinuous AJs in SCLS-*TLN1* mutant EC monolayers compared with controls. Quantification of the number and the distribution of VE-cadherin fragments showed that EC-Tln^Δex54^ ECs had significantly less large and intermediate AJs and significantly more small junctional fragments compared with control cells (Figure 3D). The decreased VE-cadherin signal in EC-Tln^Δex54^ AJs was not caused by changes in total VE-cadherin expression levels (Supplemental Figure 10) and thus indicates a functional disruption of AJ dynamics as a consequence of the mutation.

**Figure 4:**
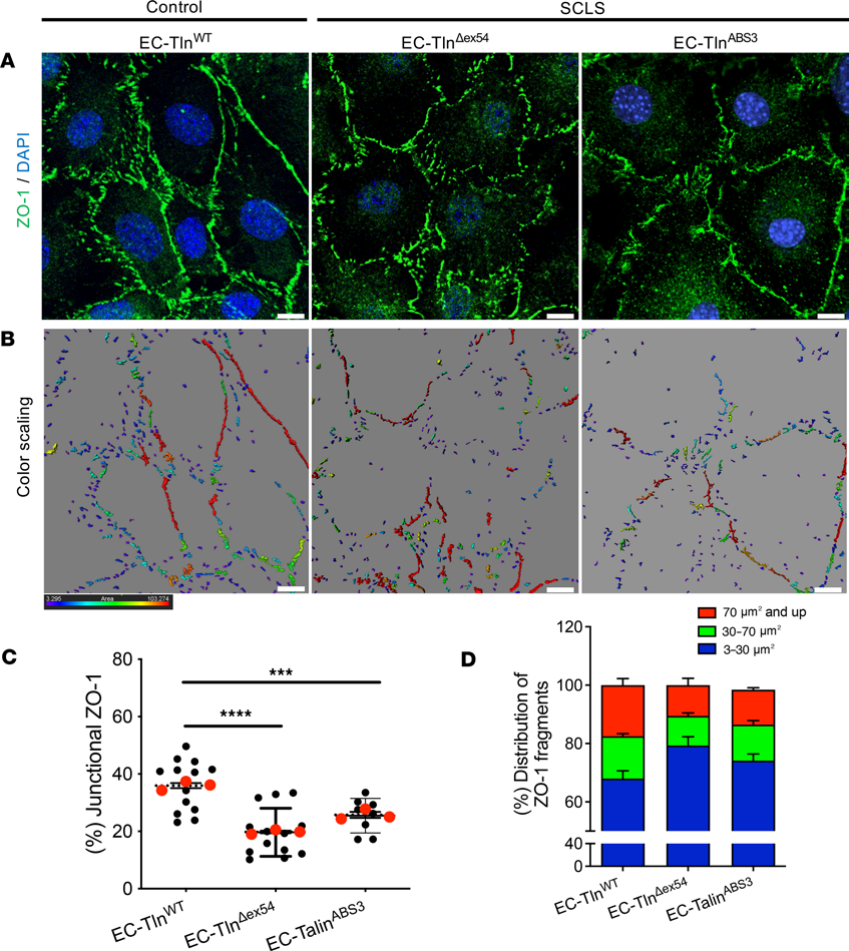
Disruption of TJs in SCLS mutant endothelial monolayers. (**A**) Representative confocal 3D images of ZO-1 (green) immunostained confluent monolayers of heterozygous-talin1 primary ECs transfected with full-length talin1 protein (EC-Tln^WT^) or SCLS-*TLN1* mutant lacking the 21 aa of exon 54 (EC-Tln^Δex54^) or the talin1 ABS3 mutation, R2510A (EC-Tln^ABS3^). Nuclei were stained with DAPI (blue). (**B**) Color scaling of the ZO-1 staining area, whereby the red color is the highest continuous staining area (>70 μm^2^), decreasing to smaller areas marked by different colors until it reaches the lowest measurements, which are of blue-violet color (<30 μm^2^), analyzed by IMARIS. Scale bars: 10 μm. (**C**) Graph displays the quantification of the continuous junctional area, as represented by the percentage of staining surfaces greater than 30 μm^2^ versus the total staining area. Data represent the mean area per field of view. *n* fields of view analyzed: EC-Tln^WT^ = 13; EC-Tln^Δex54^ = 13, EC-Tln^ABS3^ = 8. Red dots represent the mean ± SEM of 3 independent experiments. ***P = 0.0002, ****P < 0.0001 by 1-way ANOVA with Dunnett’s multiple-comparison test. (**D**) Graph displays the distribution of 3 different indexes of junctional fragments as a percentage of the total staining surfaces. Data represent the mean ± SEM of 3 independent experiments. *n* fields of view analyzed: EC-Tln^WT^ = 14; EC-Tln^Δex54^ = 11, EC-Tln^ABS3^ = 9.

Tight junctions (TJs) are also important for endothelial barrier function and are influenced by both AJs and cell-matrix adhesions (32). We next checked the TJ integrity using immunofluorescent staining of the TJ protein, ZO-1, which showed disorganized and fragmented TJs in EC-Tln1^Δex54^ compared with control EC-Tln^WT^ monolayers (Figure 4, A and B). Quantification of the junctional ZO-1 area revealed reduced localization of ZO-1 at cell-cell borders (Figure 4C). Similar to AJs, SCLS*-TLN1* mutant monolayers presented significantly less continuous TJs and smaller TJs, as shown by the quantification of distribution of junctional fragments of ZO-1 (Figure 4D).

### Mutation of the C-terminal ABS in talin reproduces the SCLS-TLN1 mutant defect.

Given that the SCLS-*TLN1* variant disrupts the R13 domain of talin, which is important for binding to actin, we next set out to examine whether disruption of the C-terminal ABS (ABS3) affects endothelial junctional integrity. We showed previously that an R2510A point mutation in ABS3 disrupts talin binding to actin without affecting talin dimerization (12) and so we used this mutant to specifically disrupt ABS3 actin binding. We transfected talin1-heterozygous ECs with a talin1 expression construct carrying the R2510A mutation fused to GFP (EC-Tln^ABS3^). We verified that mutant EC-Tln^ABS3^ protein was localized at cell-ECM adhesions similar to control full-length talin1 protein (EC-Tln^WT^) (Supplemental Figure 8). Next, we examined the morphology of AJs with the talin1 ABS3-mutant protein (EC-Tln^ABS3^) compared with EC-Tln^WT^–expressing endothelial monolayers. Similar to the SCLS-*TLN1* mutant lacking exon 54, disruption of talin binding to actin by R2510A mutation resulted in severely disrupted AJs (Figure 3, A and B), with reduced VE-cadherin localization at cell-cell borders and fragmented AJ area indicating that perturbation of actin binding to talin ABS3 reproduces the SCLS phenotype (Figure 3, C and D). As in EC-Tln^Δex54^, the total levels of VE-cadherin protein were not affected in EC-Tln^ABS3^ (Supplemental Figure 10). In support of these findings, another structural component of AJs, β-catenin, was similarly disrupted in EC-Tln^ABS3^ monolayers compared with control EC-Tln^WT^ (Supplemental Figure 11).

The ABS3 mutation also reproduced the other investigated effects of the SCLS-*TLN1* mutation. Immunofluorescent staining of ZO-1 in EC-Tln^ABS3^ ECs revealed fragmented TJs compared with control EC-Tln^WT^ (Figure 4, A and B). Quantification of ZO-1 staining showed reduced junctional ZO-1 and significantly less large and continuous TJs in EC-Tln^ABS3^ monolayers compared with control EC-Tln^WT^ (Figure 4, C and D). In agreement with the findings in patient fibroblasts, immunofluorescence analysis of active integrin β1 showed no difference between talin1-ABS3 mutant (EC-Tln^ABS3^) and control EC-Tln^WT^ ECs (Supplemental Figure 12). Taken together, these analyses suggest that the endothelial junctional disorganization caused by the SCLS-*TLN1* mutation is a result of defective binding of talin’s ABS3 to actin.

**Figure 5:**
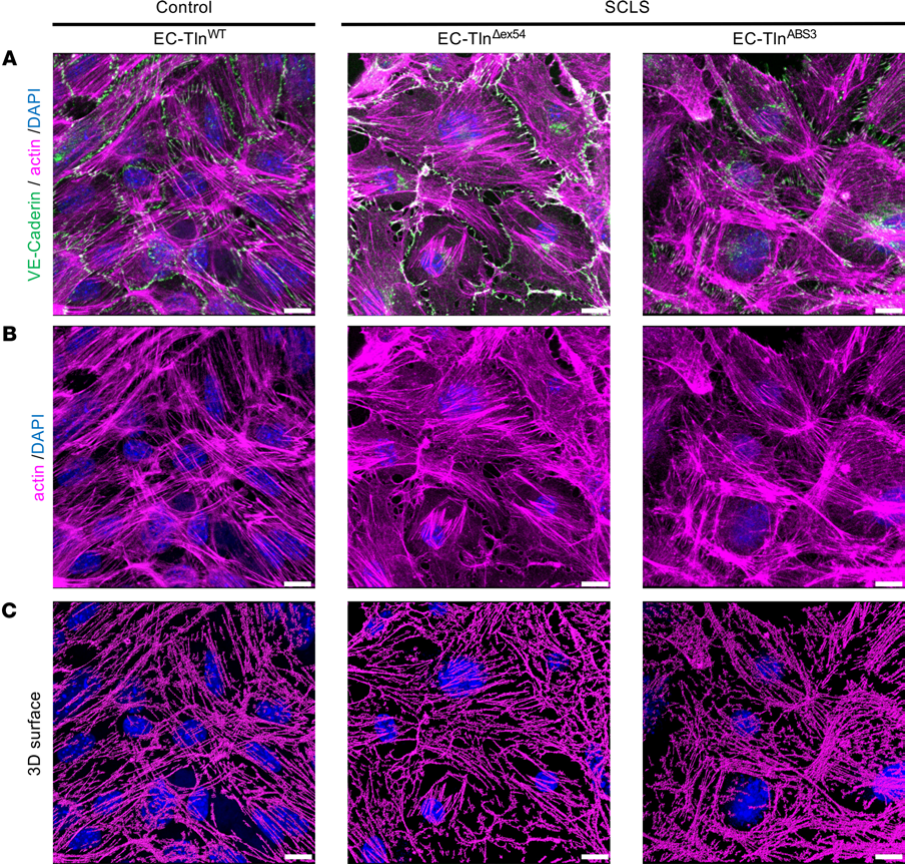
Actin cytoskeleton is not severely affected in SCLS mutant endothelial monolayers. (**A**) Representative confocal 3D images of VE-cadherin (green) and actin (magenta) immunostained confluent monolayers of heterozygous-talin1 primary ECs transfected with full-length talin1 protein (EC-Tln^WT^) or SCLS-*TLN1* mutant lacking the 21 aa of exon 54 (EC-Tln^Δex54^) or the talin1 ABS3 mutation, R2510A (EC-Tln^ABS3^). Nuclei were stained with DAPI (blue). (**B**) Single actin staining of the panels in **A**. (**C**) 3D surfaces of actin staining generated in IMARIS software. Scale bars: 10 μm. Representative images of 3 independent experiments performed with different primary EC populations.

To address this further, we examined whether the SCLS-*TLN1* mutation disrupts the actin cytoskeleton. Immunofluorescent staining by phalloidin and 3D construction of stress fiber surfaces in both SCLS-modeled (EC-Tln^Δex54^ and EC-Tln^ABS3^) and control EC-Tln^WT^ endothelial monolayers showed no significant disruption of the actin cytoskeleton (Figure 5). Therefore, we conclude that the SCLS-*TLN1* mutation and the resulting perturbation of talin’s ABS3 disrupts the endothelial junctions without affecting the actin organization under basal conditions.

### The SCLS-TLN1 mutant impairs endothelial barrier function.

**Figure 6:**
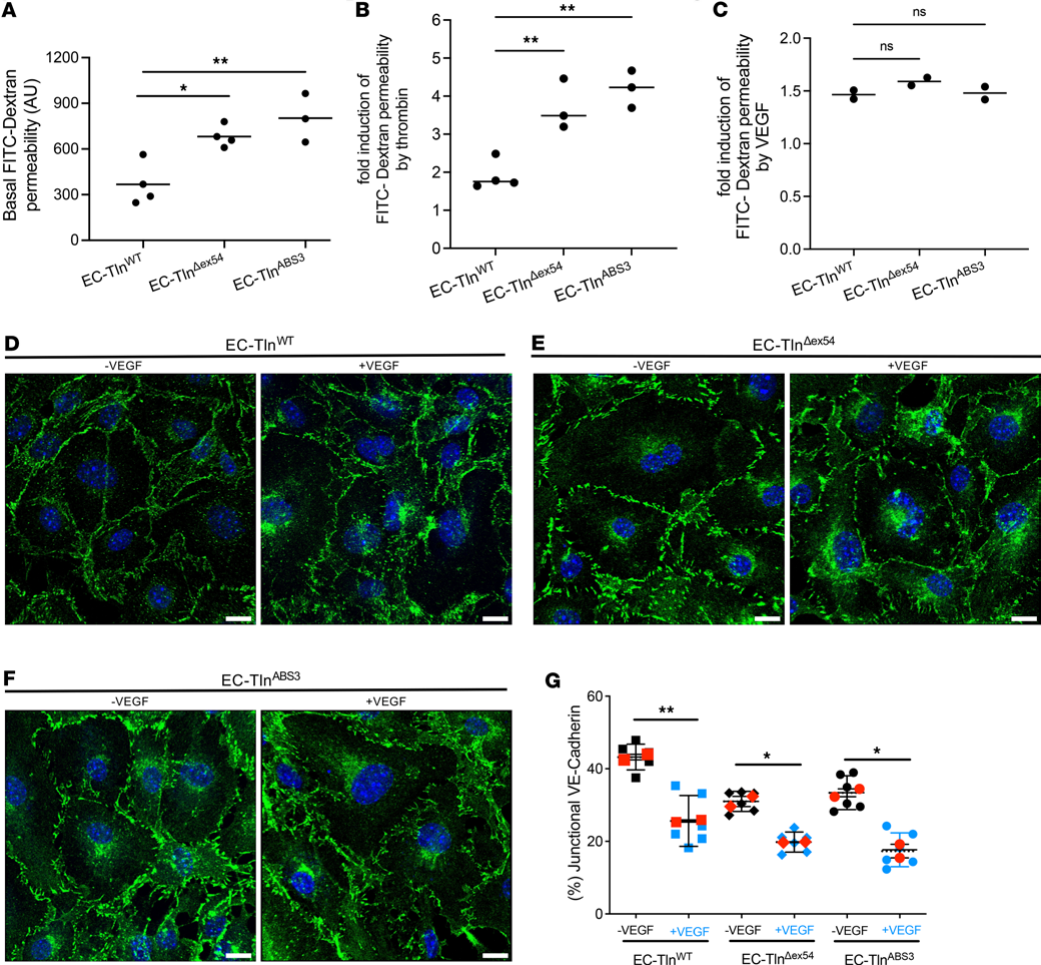
SCLS-*TLN1* mutation increases basal and agonist-induced endothelial permeability. (**A**) Basal, (**B**) thrombin-induced, and (**C**) VEGF-induced leakage of FITC-dextran through full-length WT control talin1 (EC-Tln^WT^), SCLS-*TLN1* mutant (EC-Tln^Δex54^), and talin1 ABS3 R2510A mutant (EC-Tln^ABS3^) endothelial monolayers, as measured by the Transwell assay. (**A**) Scatter plots display the values of fluorescence intensity (arbitrary units) of at least 3 independent experiments. *n* EC-Tln^WT^ = 4; *n* EC-Tln^Δex54^ = 4, *n* EC-Tln^ABS3^ = 3. (**B** and **C**) Scatter plots display the fold increase over the basal permeability in each monolayer induced by (**B**) thrombin in at least 3 independent experiments or (**C**) VEGF in 2 independent experiments. *P = 0.0136; **P = 0.0034 (**A**); **P = 0.0043; **P = 0.0011 (**B**) by 1-way ANOVA with Dunnett’s multiple-comparison test. ns, no statistical significance (**C**). (**D**–**F**) Representative confocal 3D images of VE-cadherin (green) immunostained confluent monolayers of heterozygous-talin1 primary ECs transfected with (**D**) EC-Tln^WT^ or (**E**) EC-Tln^Δex54^ or (**F**) EC-Tln^ABS3^. Nuclei were stained with DAPI (blue) Scale bars: 10 μm. (**G**) Graph displays the quantification of the continuous junctional VE-cadherin area, as represented by the percentage of staining surfaces greater than 30 μm^2^ versus the total staining area with and without VEGF stimulation. Data represent the mean area per monolayer. *n* = 6 fields of view analyzed. Red symbols represent the mean ± SEM of 2 independent experiments. *P < 0.02, **P < 0.002 by unpaired, 2-tailed *t* test.

To examine the functional impact of the disorganized AJs and TJs observed in SCLS-modeled ECs, we performed permeability assays under basal conditions. In this experiment, the leakage of FITC-dextran through an endothelial monolayer is measured, which provides a quantitative readout of monolayer integrity. Quantification of FITC-dextran leakage through endothelial monolayers demonstrated increased permeability in both EC-Tln^Δex54^ and EC-Tln^ABS3^ ECs compared with control EC-Tln^WT^ (Figure 6A). Stimulation of endothelial permeability can be further assessed by using thrombin treatment, which results in further disruption of endothelial barrier function. Both EC-Tln^Δex54^ and EC-Tln^ABS3^ monolayers displayed an approximately 4-fold increase in FITC-dextran influx upon thrombin treatment compared with a 2-fold increase in control EC-Tln^WT^ monolayers (Figure 6B).

Next, we examined the effect of SCLS-*TLN1* mutation in endothelial AJ morphology and function upon stimulation with vascular endothelial growth factor (VEGF). VEGF, initially identified as vascular permeability factor, regulates endothelial permeability and function (33). Previously, VEGF was found in the sera of episodic patients, indicating a possible role in SCLS (7). Both SCLS-modeled endothelial monolayers displayed an increase in VEGF-induced endothelial leakage that was similar to control (Figure 6C). VEGF is less potent than thrombin in disrupting the endothelial barrier (34). Therefore, the similar responses observed between SCLS-*TLN1* mutant and control endothelial monolayers are consistent with already disrupted endothelial junctions that can not be further destabilized by VEGF treatment. Immunofluorescent staining of VE-cadherin showed that VEGF stimulation generated fragmented AJs in EC-Tln^WT^ monolayers and caused further disruption of AJs in both EC-Tln^Δex54^ and EC-Tln^ABS3^ monolayers (Figure 6, D–F). Quantification of junctional VE-cadherin showed that VEGF stimulation decreased the continuous AJs, as expected by the function of VEGF in opening the endothelial barrier (Figure 6G). Interestingly, stimulation of VEGF had a less pronounced effect on the already disrupted AJs in both EC-Tln^Δex54^ and EC-Tln^ABS3^ monolayers (Figure 6, E and F). Both SCLS-modeled endothelial monolayers displayed a small decrease in junctional VE-cadherin upon VEGF treatment (Figure 6G).

### The SCLS-TLN1 mutation weakens endothelial AJs by sequestering vinculin at cell-ECM adhesions.

Finally, we set out to investigate the molecular mechanism of the endothelial barrier disruption caused by the SCLS-*TLN1* mutation. Using 2 different antibodies, one against the N-terminus and another against the C-terminus of the talin1 protein, we observed, as expected, robust staining of the cell-ECM adhesions at the bottom of the endothelial monolayers. However, we could not detect any colocalization of talin1 with VE-cadherin at AJs (Supplemental Figure 13A). In agreement, immunofluorescent staining of transiently transfected and unsorted endothelial monolayers revealed that the SCLS-*TLN1* mutant and the talin1 ABS3–mutant proteins both localized to cell-ECM adhesions and were not localized at the VE-cadherin–positive AJs (Supplemental Figure 13B). Therefore, we concluded that talin1 affects the endothelial barrier without being a structural component of cell-cell junctions.

**Figure 7:**
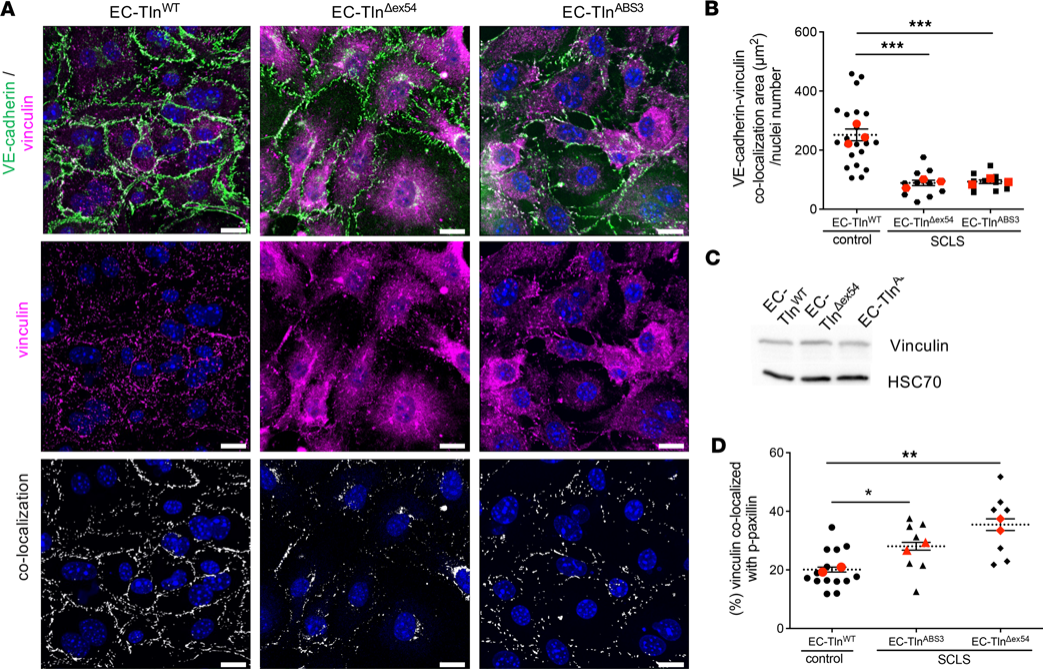
Defective vinculin-dependent stabilization of AJs in SCLS-modeled endothelial monolayers. (**A**) Representative confocal 3D images of VE-cadherin (green) and vinculin (magenta) immunostained confluent monolayers of heterozygous-talin1 ECs transfected with full-length talin 1 (EC-Tln^WT^), SCLS-*TLN1* mutant lacking the 21 aa of exon 54 (EC-Tln^Δex54^), or the talin1 ABS3 mutation (EC-Tln^ABS3^). Vinculin signal alone (magenta) and the colocalization signal of VE-cadherin/vinculin (white) are shown in the middle and bottom panels, respectively. Nuclei were stained with DAPI (blue). (**B**) Graph displays the quantification of the colocalization area of VE-cadherin and vinculin normalized to the number of nuclei present in each field of view. (**C**) Western blot analysis of total vinculin expression levels in control and SCLS-modeled ECs. HSC70 served as a loading control. (**D**) Graph displays the quantification of the percentage of vinculin colocalized with p-Y31-paxillin at dynamically remodeled adhesion sites. In all graphs, data represent the percentage mean colocalization area per image; *n* fields of view analyzed for **B**: EC-Tln^WT^ = 21; EC-Tln^ABS3^ = 11; EC-Tln^Δex54^ = 11 and for **D**: EC-Tln^WT^ = 14; EC-Tln^ABS3^ = 7; EC-Tln^Δex54^ = 7. Red symbols represent the mean ± SEM of 3 independent experiments for **B** and 2 independent experiments for **D**. ***P < 0.0003 (**B**) and *P = 0.0507; **P = 0.0085 (**D**) by 1-way ANOVA with Dunnett’s multiple-comparison test. Scale bars: 10 μm.

Talin has multiple binding sites for vinculin, a key adaptor that can bind to both talin and the actin cytoskeleton and thus can facilitate adhesion stability (13, 35, 36). Additionally, vinculin has been shown to be an important regulator of barrier function both in epithelial cells and ECs (37–39). Therefore, we examined whether the SCLS-*TLN1* mutation affects vinculin localization at cell-cell junctions. Immunofluorescence analysis of vinculin staining revealed a significant reduction in vinculin colocalization with VE-cadherin in EC-Tln^Δex54^ monolayers compared with the EC-Tln^WT^ control (Figure 7, A and B). The decreased vinculin localization at cell-cell junctions was also observed with the EC-Tln^ABS3^ ECs, further supporting the similar function of the SCLS and actin-binding mutants (Figure 7, A and B). The disrupted localization of vinculin at AJs in both SCLS-modeled ECs was not caused by reduced total levels of vinculin expression, as these appeared unchanged by Western blot analysis (Figure 7C).

Increased mechanical forces and actomyosin contractility drive the translocation of vinculin to cell-ECM adhesions (38, 40). Therefore, we next examined whether the SCLS-*TLN1* mutant affects the localization of vinculin at cell-ECM adhesions. In both SCLS-modeled endothelial monolayers, we found vinculin to be enriched in actively remodeled cell-ECM adhesions, as demonstrated by the significantly increased colocalization of vinculin with p-Y31-paxillin (Figure 7D and Supplemental Figure 14A). The total expression levels of paxillin remained unchanged, as shown by Western blot analysis (Supplemental Figure 14B).

We propose a model to explain the disruption of endothelial barrier stability observed in SCLS based on changes in vinculin localization. It has been shown previously that vinculin is responsible for stabilizing AJs, without affecting their formation (37, 41). Vinculin also translocates to cell-ECM adhesions upon increased mechanical forces and can strengthen cell-ECM adhesions by binding to actin filaments (35, 38, 40, 42–44). The interactions of talin and vinculin are tightly regulated and the force loading on talin is critical to this process. We propose that in SCLS, disturbance of talin’s actin-binding capabilities results in accumulation of vinculin in the remodeling cell-ECM adhesions possibly by dysregulation of the force loading on talin. The cellular levels of vinculin are limiting and increased localization of vinculin at cell-ECM adhesions hinders its translocation to AJs. Reduction of vinculin in AJs leads to less stable cell-cell junctions. Therefore, when a trigger induces endothelial permeability, AJs are unable to be remodeled properly and timely, causing vascular leakage, which is the hallmark of SCLS.

## Discussion

In this study, we report a familial case of SCLS with 3 affected individuals from an extended pedigree with SCLS who each carry a rare heterozygous splice-site variant in the *TLN1* gene. This splice variant distorts the R13 talin domain and is predicted to affect the C-terminal ABS, ABS3. Talin1 protein is a key cell-ECM adhesion component that can activate integrins, sense and respond to mechanical forces, and mediate the link to the actin cytoskeleton (11, 45, 46). Given that the patient fibroblasts had unaltered cell-ECM adhesions and integrin functions and that talin has established roles in vascular function (16–18), we modeled the effect of the SCLS-*TLN1* variant in ECs heterozygous for talin1. We found that the deletion of 21 amino acids from the talin1 protein as a result of the exon skipping severely impaired endothelial barrier integrity and function. We show that this impairment is a result of disrupted AJ and TJ stability. Similarly, a mutation that specifically perturbed the ability of talin’s C-terminus to bind actin had similar effects. Strikingly, we found that both mutations resulted in vinculin being sequestered at the cell-ECM adhesions, concomitant with depletion of vinculin from AJs. Recently, it was reported that EC-specific deletion of talin1 decreases VE-cadherin expression and localization at AJs, causing endothelial barrier dysfunction and bleeding (18, 19). In contrast with a complete endothelial talin1 deletion, the heterozygous SCLS-*TLN1* mutations had no effect on VE-cadherin protein expression, but severely disturbed its localization at cell-cell contacts, resulting in fragmented AJs.

**Figure 8:**
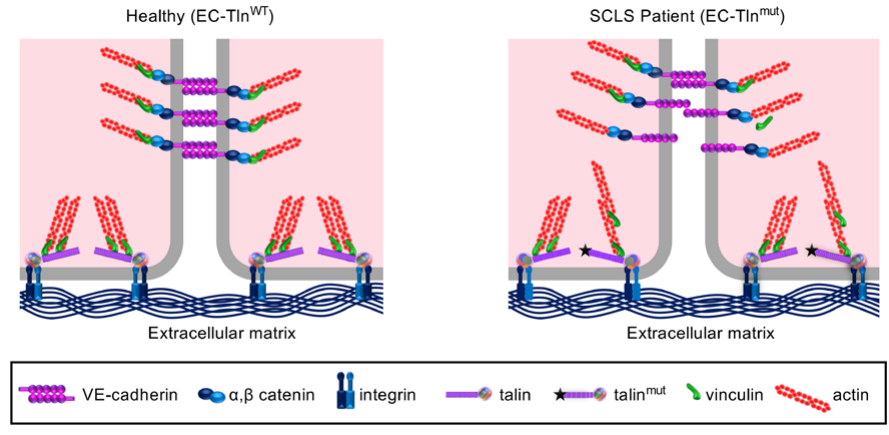
Schematic model of how the SCLS-*TLN1* mutation affects endothelial barrier function. In WT ECs, vinculin is dynamically distributed between both cell-ECM and cell-cell adhesions to regulate their dynamics. In SCLS-*TLN1* mutant ECs with heterozygous disruption of talin1 R13 domain, the vinculin localization at adherens junctions is severely impaired, leading to defective endothelial barrier function. The disorganization of adherens junctions observed in the SCLS-*TLN1* ECs is reproduced by a talin1 mutant with defective ABS3 binding to actin. Therefore, we propose that the SCLS-*TLN1* mutant destabilizes endothelial adherens junctions by perturbing the force loading on talin. This in turn results in sequestering of vinculin at cell-ECM adhesions, depleting it from adherens junctions, which leads to defective remodeling of the cell-cell junctions.

Although the trigger for SCLS remains unknown, both inflammatory mediators and vasodilators have been found in serum from patients under attack and are believed to trigger the opening of capillary junctions and to increase permeability (6, 7, 47). Permeability agonists, among them thrombin and histamine, induce Rho-mediated actomyosin contractility and cause force-dependent remodeling of cell junctions, creating jacked AJs (41, 48, 49). Talin has been shown to be a key mechanotransducer in vitro (50) and in vivo (18) and in response to mechanical forces generated by the actomyosin contractility, talin undergoes intramolecular structural rearrangements that reveal more binding sites for interacting partners, such as vinculin (11). Recently developed FRET probes have detailed talin’s conformational changes in response to altered tension at cell-ECM adhesions and have shown that ABS3 is important for reinforcing cell-ECM adhesions (35, 51, 52). Our data suggest that the SCLS-*TLN1* variant disrupts talin1’s ABS3, creating a dysfunctional link to the actin cytoskeleton that causes vinculin to accumulate at cell-ECM adhesions (Figure 8). In agreement with this, a linear correlation between mechanical forces and residence time for vinculin at cell-ECM adhesions has been reported (53). Furthermore, permeability agonists, including thrombin, induce the translocation of vinculin to cell-ECM adhesions and these effects are mediated by increased actomyosin contractility (38, 40, 54). Consistent with this, thrombin caused a significant increase in endothelial permeability in both SCLS-modeled ECs compared with control ECs. The sequestration of vinculin at cell-ECM adhesions of the heterozygous SCLS mutants significantly depleted vinculin from AJs, which impaired vinculin’s function at AJs. This impaired response would delay the proper remodeling of cell-cell junctions after a trigger, impacting on the protection of the endothelial barrier function and its integrity.

The intermittent presentation of SCLS is intriguing and may be due to an altered percentage of mis-spliced versus WT *TLN1* transcripts under various cellular conditions as a result of a currently unidentified environmental trigger. While the trigger is still not established, a commonly reported condition prior to an SCLS episode was dehydration and identification of the trigger and the mechanism of action might indicate pathways involved in *TLN1* gene processing. VEGF has been reported to be elevated in the serum of episodic SCLS patients and suggested to contribute to the transient contraction of vascular endothelium (6, 7). In agreement with this, VEGF induced permeability in SCLS-modeled endothelial monolayers, substantiating its role in SCLS disease manifestation. Similarly, a trigger might affect the mechanical properties of the vascular endothelium. Talin tension has been reported to regulate local actin organization and focal adhesion dynamics (51). Our data suggest that talin’s ABS3 could be important for sensing endothelial tension and regulating vinculin localization and vascular barrier function. A potential therapeutic avenue might be to modulate and restore normal splicing by use of splice-switching antisense oligonucleotides (55).

In conclusion, our data demonstrate an undescribed role for the talin1 C-terminal ABS3 as a regulator of endothelial barrier stability. Most importantly, these findings are directly relevant to human pathology. Understanding the molecular mechanism of SCLS and its triggers will allow for early detection of attacks and possible preventative measures and can lead to more effective focused treatments.

## Methods

### Sex as a biological variable.

For the human studies, the sex(es) involved are specified. In the isolation of primary ECs from transgenic mice, both sexes were utilized.

### Exome analysis.

Following informed consent, exome analysis was performed on DNA extracted from whole blood of 3 affected patients and healthy control relatives. Exonic sequences from DNA were enriched with the xGen Exome Research Panel IDT-V2 Kit together with the xGen Human mtDNA Research Panel v1.0 kit. Sequences were generated on a NovaSeq 6000 sequencing system (Illumina). Read alignment and variant calling were performed with DNAnexus using default parameters with the human genome assembly hg19 (GRCh37) as reference.

### Segregation analysis.

An amplicon containing the *TLN1* variant was amplified by conventional PCR of genomic DNA isolated from members of the extended family using the following primers: TLN1_DNA_F1: 5′-GGCCTAAAGCAGGGAGAGTT-3′ and TLN1_DNA_R1: 5′-GGACAGGTTGGCACTTTGAT-3′. Amplicons were analyzed by Sanger dideoxynucleotide sequencing.

### RNA isolation and reverse transcription PCR.

RNA was isolated from peripheral blood or cultured fibroblasts of 3 patients and unaffected relative control by TRIzol reagent extraction using the Qiagen RNeasy kit according to the manufacturer’s instructions. cDNA was prepared from 1 μg RNA using a qScript cDNA Synthesis Kit (Quantabio or the RevertAid M-MuLV Reverse Transcriptase kit [Fermentas]). The region encompassing exons 52–57 of *TLN1* was amplified by a PCR reaction using PCRBIO HS Taq Mix Red (PCR Biosystems) with primers TLN1_RNA_F1: 5′-CAGAGGACCCCACAGTCATT-3′ and TLN1_RNA_R1: 5′-TCCGAAGCATTTCTTCCTGT-3′. The resultant fragments were separated by 3% (w/v) agarose gel electrophoresis and their sequence determined by Sanger sequencing. Quantitative real-time PCR was performed using primer TlnF1 (exon 54): 5′-CAATGCACTGGACGATGGG-3′ and TlnR1 (exon 55): 5′-TGCCTCACACAGATTGTTGGT-3′ on a StepOnePlus system (Applied Biosystems) and results were analyzed with the StepOne software. The human *RPLP1* gene was used as a reference gene and was amplified with primers F: 5′-AAGCAGCCGGTGTAAATGTTGAGC-3′ and R: 5′-CATTGCAGATGAGGCTCCCAATGT-3′.

### Protein analysis.

Structural modeling of the exon-skipping event was done comparing the solution structure of R13 (12) with structural models of WT-R13 and SCLS-R13 generated using the open-source software ColabFold to run AlphaFold2 software (23, 56). Structures were visualized using PyMOL (version 2.0, Schrödinger, LLC).

### Protein expression and purification.

Synthetic genes of the murine SCLS-R13-DD (residues 2300–2541, Δ2376–2395) and SCLS-R12-R13-DD (residues 2138–2541, Δ2376-2395) in the pET151 expression vector were purchased from GeneArt. The equivalent WT constructs were already available and described previously (12). Recombinant His-tagged chicken Vd1 (residues 1–258) was expressed from a pET-15b plasmid. The 5 constructs were each expressed in BL21(DE3) *E*. *coli* cells grown in lysogeny broth (LB) with 100 μg/mL ampicillin at 37°C. Protein expression was induced with 0.2 mM isopropyl β-D-1-thiogalactopyranoside (IPTG) overnight at 18°C. Proteins were purified by nickel affinity chromatography, followed by anion exchange as described previously (57). R12-R13-DD and SCLS-R12-R13-DD were spun on their own at 50,000*g* for 20 minutes at 4°C. The samples were analyzed using SDS-PAGE.

### SEC.

Analysis of talin1 R12-R13-DD and SCLS-R12-R13-DD alone and in the presence of Vd1 was performed by SEC at room temperature. Samples were run at 100 μM talin and 100 μM Vd1. The samples were loaded and run using a Superdex 200 Increase 10/300 GL (Cytiva).

### CD.

CD experiments were performed using a JASCO J-715 spectropolarimeter. Samples were 0.5 mg/mL R12-R13-DD WT and R12-R13-DD SCLS in 20 mM Tris pH 8, 150 mM NaCl. Far-UV spectra were recorded at wavelengths 200–260 nm, using 6 scans at 50 nm/min speed, 1 nm step resolution, and 1 nm band width. Melting curves were collected at a fixed, 222 nm wavelength, varying the temperature between 20°C and 90°C, with 1°C step resolution and 1 nm band width.

### Primary cell cultures.

Primary fibroblasts were isolated from skin samples of patients IV-3, IV-11, and healthy relatives. Cells were cultured in DMEM supplemented with 10% FCS and 1% penicillin/streptomycin and L-glutamine. Early cell passages (P2–P8) were used for all experiments.

Mouse primary ECs were isolated from lungs of *TLN1*-heterozygous (*pdgfBB*iCre-ER^T2^ *TLN1^fl/+^*) or WT control mice, as described previously (58). All mice used were on the C57BL/6J background, backcrossed 10 times, both sexes, aged 6–8 weeks old. Briefly, lungs were minced, digested with 0.1% collagenase type I for 1 hour, passed through a 70 μm pore size cell strainer, and after centrifugation cells were resuspended in mouse lung endothelial cell medium (Ham’s F-12/DMEM 1:1, 1% penicillin/streptomycin and L-glutamine, 20% FCS and endothelial mitogen), and plated onto tissue-culture flasks precoated with a mixture of collagen I, human plasma fibronectin, and 0.1% gelatin. ECs were purified by magnetic immunosorting using anti–ICAM-2 (1:300; BD Biosciences, 553325) and anti–PECAM-1 (1:1000; BD Biosciences, 553370) antibodies. Cultures were tested for endothelial purity by flow cytometric analysis using PECAM-1, VE-cadherin, and ICAM-2 antibodies. All experiments were performed in 97%–99% pure ECs, in early passages (P2–P6) of pure ECs.

### Live cell imaging.

To assess cell spreading, control and patient fibroblasts were seeded on coated wells of 6-well tissue culture plates and transferred to an environmental chamber at 37°C and 5% CO_2_ coupled to a Zeiss Observer Z.1 microscope. Two different planes were chosen from each well and photos were acquired every 5 minutes for 72 cycles. Camera and shutter were controlled by the Zen software (Zeiss) and movies were processed using the same program.

### Primary EC transfections.

Primary ECs were treated with 4-hydroxy-tamoxifen (10 μM) for 72 hours and transfected with GFP-talin1-WT (EC-Tln^WT^), GFP-talin1-Δexon 54 (EC-Tln^Δex54^), or GFP-talin1-R2510A (EC-Tln^ABS3^) constructs using the Amaxa Nucleofector kit (VP1-1001, Lonza) or the NEPA21 super electroporator (NEPAGENE), according to the manufacturers’ instructions. After transfection, cells were seeded in 100-mm precoated tissue culture plates and left to recover overnight. Thirty hours after transfection, ECs were sorted by flow cytometry (FACSAria II, BD Biosciences) and only the GFP-positive cells were kept, which were afterwards seeded in 8-well ibidi slides for further immunofluorescence analysis or plated for permeability assays.

### Immunofluorescence analysis of cell-ECM adhesions.

For the analysis of focal adhesion sites mouse primary ECs (either WT or transfected with the mutant talin constructs) or primary fibroblasts were seeded on 13-mm round glass coverslips in 24-well culture plates at a density of 2 × 10^4^ cells/well. After 48 hours, cells were fixed with 4% paraformaldehyde for 10 minutes at 4°C, washed 3 times in 1× PBS, and then permeabilized with 0.3% Triton X-100/PBS for 10 minutes. Afterwards, cells were washed once with PBS and incubated for 1 hour at room temperature in 1% BSA/PBS for blocking. Primary antibodies anti-talin1 (clone 97H6, Bio-Rad, MCA4770, 1:500), anti-talin2 (custom made, 1:50; ref. 16), anti-paxillin (BD Biosciences, 610052, 1:200), anti–p-Tyr118-paxillin (Cell Signaling Technology, 2541, 1:200), anti-vinculin (Sigma-Aldrich, V9264, 1:400), anti–p-Tyr397-FAK (Biosource, 44624G, 1:100), or anti–integrin-β1 (clone 9EG7, BD Biosciences, 550531) were added on the coverslips in 0.1% BSA/PBS blocking buffer and incubated overnight at 4°C. The next day, cells were washed 3 times with PBS and incubated with the appropriate secondary antibodies (Alexa Fluor conjugated, Thermo Fisher Scientific, 1:400) and phalloidin-633 for F-actin (Thermo Fisher Scientific, A-22284, 1:400) for 1 hour at room temperature in 1% BSA/PBS, where 4′,6-diamidino-2-phenylindole (DAPI) was also added. After 3 final washes, coverslips were mounted on microscope slides with Mowiol. Images of *Z*-stacks were captured using a Zeiss Observer Z.1 or a confocal SP8X WLL system (Leica Microsystems) and quantification of the focal adhesions on the rendered *Z*-stacks was performed with IMARIS software (Oxford Instruments).

### Immunofluorescence analysis of cell-cell junctions.

Mouse primary ECs were seeded on 8-well coated μ-slides (ibidi) at a density of 5 × 10^5^ cells/well and incubated for 48–52 hours to form junctions. Afterwards, they were fixed using 4% paraformaldehyde for 10 minutes at 4°C and processed for immunofluorescent staining as described above. In the final step, cells were kept in PBS solution and imaged directly from the 8-well slides. Antibodies used were anti–VE-cadherin (clone 11D4, BD, 555289, 1:100), anti–ZO-1 (clone 1A12, Thermo Fisher Scientific, 339100, 1:100), anti–β-catenin (clone 15B8, Sigma-Aldrich, 1:1000), anti-talin1 (clone 97H6, Bio-Rad, MCA4770, 1:500), anti-talin1-TD77 (Merck-Millipore, 05-1144, 1:100), anti-vinculin (Sigma-Aldrich, V9264, 1:400), anti–p-paxillin (Cell Signaling Technology, 2541, 1:200), anti-actin (Sigma-Aldrich, A2103, 1:100), and phalloidin-633 for F-actin (Thermo Fisher Scientific, A-22284, 1:400). All secondary antibodies were Alexa Fluor conjugated (Thermo Fisher Scientific) and were used at a 1:400 dilution. Images of *Z*-stacks were acquired using a confocal SP8X WLL system (Leica Microsystems) and analyzed with IMARIS software (Oxford Instruments). For quantification of the junctional integrity, the VE-cadherin, ZO-1, or β-catenin signal from the confocal *Z*-stacks was translated into 3D surface objects, which were further color coded and categorized according to their sizes through the vantage plot function. The area of each object was used to measure the fragmented cell junctions and quantify their distribution.

### Western blot analysis.

For protein level analysis, mouse primary ECs or human primary fibroblasts were either lysed directly or were serum starved overnight (in DMEM with 1% FCS) and then serum-stimulated (in DMEM with 10% FCS) for 10 minutes before lysis. Lysis buffer consisted of 3% SDS, 60 μM sucrose, 65 mM Tris-HCl pH 6.8 supplemented with protease and phosphatase inhibitors (Roche). Protein concentration was determined using the BSA Protein Assay kit (Thermo Fisher Scientific) and 30–60 μg of protein from each sample was loaded onto 10%–12% polyacrylamide gels. After electrophoresis, proteins were transferred to PVDF membranes, blocked using 5% milk in Tris-buffered saline with 0.1% Tween 20 (TBS-T), and incubated overnight with the primary antibodies diluted in 3% BSA/TBS-T at 4°C. The next day, blots were washed 3 times with TBS-T and then incubated with the relevant HRP-conjugated antibodies in 5% milk/TBS-T for 1 hour at room temperature. After another 3 washes, chemiluminescence was detected using the Luminata Crescendo Western HRP substrate and a ChemiDoc XRS+ imaging system (Bio-Rad). Densitometry readings were acquired using Image Lab software. The antibodies used included anti–p-Ser473-AKT, anti-AKT, anti–p-p44/42 MAPK, anti-p44/42 MAPK, anti–p-Tyr118-paxillin (all 1:1000; Cell Signaling Technology, 4060, 4685, 9101, 9102, and 2541), anti–p-Tyr397-FAK (Thermo Fisher Scientific, 44624G), anti-talin1 (clone 97H6, Bio-Rad, MCA4770, 1:10,000), anti-talin2 (1:200, custom made), anti–VE-cadherin (BD Biosciences, 555289, 1:1000), anti–integrin-β1 (Abcam, ab3167, 1:1000); anti-vinculin (Sigma-Aldrich, V9264, 1:1000), and anti-HSC70 (Santa Cruz Biotechnology, sc7298, 1:5000). The appropriate HRP-conjugated anti-mouse, anti-rabbit, or anti-rat antibodies (1:5000, Jackson Immunoresearch, 115-035-008, 115-035-046, and 115-036-03) were used as secondary antibodies.

### Flow cytometry.

For integrin-β1 analysis, control and patient fibroblasts were harvested by trypsinization, washed, and then treated with either PBS alone or PBS supplemented with 1 mM MnCl_2_ for 20 minutes. After 2 washes, cells were incubated with primary antibodies against total integrin-β1 (Abcam, ab3167) or integrin-β1 (clone 9EG7, BD Biosciences, 550531) diluted 1:50 in FACS buffer (2% FCS in PBS with 0.05% sodium azide) for 45 minutes at 4°C. After another 2 washes, cells were incubated with the secondary antibodies (Alexa Fluor 488 anti-rat or anti-mouse, Thermo Fisher Scientific, A-11017 and A-11006) in FACS buffer for 30 minutes on ice, washed again, and resuspended in PBS to be analyzed on a BD FACSCanto II instrument with FlowJo software.

### Permeability assay.

FACS-isolated GFP-positive transfected ECs were seeded at a density of 2 × 10^5^ cells per well in 6.5-mm Transwells with 0.4-μm pore polyester membrane-insert plates (Corning, 3470) precoated with collagen I and fibronectin and left for 48–52 hours to form cell junctions. The bottom chamber contained 400 μL Opti-MEM (Gibco, 11058-021). The integrity of the monolayer was examined in every step using phase-contrast microscopy. FITC-dextran (Sigma-Aldrich, FD2000S) solution at a concentration of 1 mg/mL was added to the upper chamber of the Transwell and incubated for 1 hour. The fluorescence was measured in 100 μL collected from the bottom chamber of the Transwell in a 96-well plate using a photometer (TECAN Infinite M200) with excitation 490 nm and emission 520 nm. Measurements were performed in duplicate, using optimal settings available with the software. Endothelial permeability was induced by incubating endothelial monolayers, previously starved for 1 hour in Opti-MEM, with 0.2 μg/mL thrombin (GE Healthcare, 27-0846-01) for 1 hour. For VEGF-induced permeability, endothelial monolayers were measured under basal conditions and starved for 30 minutes in Opti-MEM before treatment with 100 ng/mL mouse VEGF 165 (Peprotech, 450-32) for 1 hour. FITC-dextran from the bottom well of an empty Transwell, not containing a cell monolayer, was measured in every experiment, as overflow control.

### Platelet adhesion tests.

Platelet adhesion was measured by the cone and plate(let) analyzer (CPA) as previously described (59). Briefly, whole blood was placed in polystyrene plates and subjected to a shear rate of 1,800 s^–1^ using a rotating Teflon cone. The plates were then thoroughly washed with distilled water, stained, and analyzed with an inverted microscope connected to an image analysis system. Two parameters of platelet adhesion were evaluated: percentage of platelet surface coverage (SC, %) and the average size (AS, μm^2^) of the platelet aggregates bound to the surface.

### Statistics.

GraphPad Prism software (v10.0) was used for statistical analysis. All data are presented as mean ± SEM for at least 2–3 independent experiments, as stated in the corresponding figure legends. Statistical significance was determined by unpaired, 2-tailed *t* test or 1-way ANOVA with Dunnett’s multiple-compassion test on the means from different experiments, as stated in the figure legends. A *P* value of less than 0.05 was considered significant. The relevant *P* values are indicated in each legend.

### Study approval.

All human studies were in compliance with ethical standards. Written informed consent was obtained in accordance with IRB-approved protocol 0306-10-HMO. All experimental protocols involving animals were approved by the Veterinary Administration Bureau, Prefecture of Athens, Greece, under compliance to the national law and the EU Directive 63/2010 and performed in accordance with the guidance of the Institutional Animal Care and Use Committee of BSRC Alexander Fleming.

### Data availability.

The ClinVar accession number for the DNA variant data reported in this manuscript is SCV002525869. Raw data supporting the conclusions of this study can be found in the Supporting Data Values file.

## Author contributions

NE reported the proband IV-3, collected clinical data, analyzed genomic data, and wrote the manuscript. GR generated the SCLS-*TLN1* mutant ECs, performed experiments, analyzed the EC junctions, and wrote the manuscript. DOL performed exome sequencing and analyzed genomic data. CA performed studies of patient fibroblasts and quantitative RT-PCR experiments. SYD performed molecular studies on patient-derived samples. ARC and WJSE performed biochemical and structural analyses. NJB generated the GFP-talin1-Δexon 54 construct. VM analyzed exome sequencing data. RSS reported the proband IV-11 and collected clinical data. VV collected clinical data. SG performed FACS and provided advice on FACS analysis. DL oversaw genetic analysis and data interpretation. BTG performed biochemical and structural analyses, interpreted data, and wrote and revised the manuscript. TH oversaw clinical and genetic data interpretation and revised the manuscript. VK conceptualized the study, designed and performed experiments, analyzed and interpreted data, acquired funding, and wrote and revised the manuscript.

## Supplementary Material

Supplemental data

Unedited blot and gel images

Supplemental video 1

Supporting data values

## Version 1 12/20/2024

Electronic publication

## References

[1] Clarkson B (1960). Cyclical edema and shock due to increased capillary permeability. Trans Assoc Am Physicians.

[2] Marks J, Shuster S (1973). Disorders of capillary permeability. Br J Dermatol.

[3] Amoura Z (1997). Systemic capillary leak syndrome: report on 13 patients with special focus on course and treatment. Am J Med.

[4] Eo TS (2018). Clinical presentation, management, and prognostic factors of idiopathic systemic capillary leak syndrome: a systematic review. J Allergy Clin Immunol Pract.

[5] Xie Z (2015). High-dose intravenous immunoglobulin therapy for systemic capillary leak syndrome (Clarkson disease). Am J Med.

[6] Xie Z (2014). Inflammatory markers of the systemic capillary leak syndrome (Clarkson disease). J Clin Cell Immunol.

[7] Xie Z (2012). Vascular endothelial hyperpermeability induces the clinical symptoms of Clarkson disease (the systemic capillary leak syndrome). Blood.

[8] Xie Z (2014). Genome-wide SNP analysis of the systemic capillary leak syndrome (Clarkson disease). Rare Dis.

[9] Pierce R (2019). Whole-exome sequencing of adult and pediatric cohorts of the rare vascular disorder systemic capillary leak syndrome. Shock.

[10] Calderwood DA (2013). Talins and kindlins: partners in integrin-mediated adhesion. Nat Rev Mol Cell Biol.

[11] Goult BT (2018). Talin as a mechanosensitive signaling hub. J Cell Biol.

[12] Gingras AR (2008). The structure of the C-terminal actin-binding domain of talin. EMBO J.

[13] Goult BT (2013). RIAM and vinculin binding to talin are mutually exclusive and regulate adhesion assembly and turnover. *J Biol*. Chem.

[14] Haining AWM (2016). Talin: a mechanosensitive molecule in health and disease. FASEB J.

[15] Nikolopoulou PA (2021). The adhesome network: key components shaping the tumour stroma. Cancers (Basel).

[16] Monkley SJ (2011). Endothelial cell talin1 is essential for embryonic angiogenesis. Dev Biol.

[17] Pulous FE (2020). Talin-dependent integrin activation is required for endothelial proliferation and postnatal angiogenesis. Angiogenesis.

[19] Pulous FE (2019). Talin-dependent integrin activation regulates VE-cadherin localization and endothelial cell barrier function. Circ Res.

[20] Wei X (2017). Downregulation of Talin-1 expression associates with increased proliferation and migration of vascular smooth muscle cells inaortic dissection. BMC Cardiovasc Disord.

[21] Turley TN (2019). Rare missense variants in TLN1 are associated with familial and sporadic spontaneous coronary artery dissection. Circ: Genom Prec Med.

[22] Jaganathan K (2019). Predicting splicing from primary sequence with deep learning. Cell.

[23] Jumper J (2021). Highly accurate protein structure prediction with AlphaFold. Nature.

[24] Papagrigoriou E (2004). Activation of a vinculin-binding site in the talin rod involves rearrangement of a five-helix bundle. EMBO J.

[25] Maquat LE, Carmichael GG (2001). Quality control of mRNA function. Cell.

[26] Zhang X (2008). Talin depletion reveals independence of initial cell spreading from integrin activation and traction. Nat Cell Biol.

[27] Liu J (2015). Talin determines the nanoscale architecture of focal adhesions. Proc Natl Acad Sci U S A.

[28] Nieswandt B (2007). Loss of Talin1 in platelets abrogates integrin activation, platelet aggregation, and thrombus formation in vitro and in vivo. J Exp Med.

[29] Petrich BG (2007). Talin is required for integrin-mediated platelet function in hemostasis and thrombosis. J Exp Med.

[30] Monkley SJ (2011). Talin-dependent integrin activation is required for fibrin clot retraction by platelets. Blood.

[31] Dejana E, Orsenigo F (2013). Endothelial adherens junctions at a glance. J Cell Sci.

[32] Tornavaca O (2015). ZO-1 controls endothelial adherens junctions, cell-cell tension, angiogenesis, and barrier formation. J Cell Biol.

[33] Vandenbroucke E (2008). Regulation of endothelial junctional permeability. Ann N Y Acad Sci.

[34] Apte RS (2019). VEGF in signaling and disease: beyond discovery and development. Cell.

[35] Atherton P (2015). Vinculin controls talin engagement with the actomyosin machinery. Nat Commun.

[36] Gingras AR (2005). Mapping and consensus sequence identification for multiple vinculin binding sites within the talin rod. J Biol Chem.

[37] Huveneers S (2012). Vinculin associates with endothelial VE-cadherin junctions to control force-dependent remodeling. J Cell Biol.

[38] Birukova AA (2016). Dual role of vinculin in barrier-disruptive and barrier-enhancing endothelial cell responses. Cell Signal.

[39] Twiss F (2012). Vinculin-dependent Cadherin mechanosensing regulates efficient epithelial barrier formation. Biol Open.

[40] Birukova AA (2016). Selective role of vinculin in contractile mechanisms of endothelial permeability. Am J Respir Cell Mol Biol.

[41] Timmerman I (2015). A local VE-cadherin and Trio-based signaling complex stabilizes endothelial junctions through Rac1. J Cell Sci.

[42] Atherton P (2016). Mechanosensitive components of integrin adhesions: role of vinculin. Exp Cell Res.

[43] Stutchbury B (2017). Distinct focal adhesion protein modules control different aspects of mechanotransduction. J Cell Sci.

[44] Humphries JD (2007). Vinculin controls focal adhesion formation by direct interactions with talin and actin. J Cell Biol.

[45] Critchley DR (2005). Genetic, biochemical and structural approaches to talin function. Biochem Soc Trans.

[46] Critchley DR, Gingras AR (2008). Talin at a glance. J Cell Sci.

[47] Hsu P (2015). Idiopathic systemic capillary leak syndrome in children. Pediatrics.

[48] Dorland YL, Huveneers S (2016). Cell-cell junctional mechanotransduction in endothelial remodeling. Cell Mol Life Sci.

[49] Oldenburg J (2015). VASP, zyxin and TES are tension-dependent members of focal adherens junctions independent of the α-catenin-vinculin module. Sci Rep.

[50] Elosegui-Artola A (2016). Mechanical regulation of a molecular clutch defines force transmission and transduction in response to matrix rigidity. Nat Cell Biol.

[51] Kumar A (2018). Local tension on talin in focal adhesions correlates with F-actin alignment at the nanometer scale. Biophys J.

[52] Kumar A (2016). Talin tension sensor reveals novel features of focal adhesion force transmission and mechanosensitivity. J Cell Biol.

[53] Dumbauld DW (2013). How vinculin regulates force transmission. Proc Natl Acad Sci U S A.

[54] Birukova AA (2009). Endothelial permeability is controlled by spatially defined cytoskeletal mechanics: atomic force microscopy force mapping of pulmonary endothelial monolayer. Nanomedicine.

[55] Havens MA, Hastings ML (2016). Splice-switching antisense oligonucleotides as therapeutic drugs. Nucleic Acids Res.

[56] Mirdita M (2022). ColabFold: making protein folding accessible to all. Nat Methods.

[57] Khan RB (2020). Biochemical characterization of the integrin interactome. Methods Mol Biol.

[58] Kostourou V (2013). FAK-heterozygous mice display enhanced tumour angiogenesis. Nat Commun.

[59] Savion N, Varon D (2006). Impact – the cone and plate(let) analyzer: testing platelet function and anti-platelet drug response. Pathophysiol Haemo T.

