# Supplementary material for: Talin1 dysfunction is genetically linked to systemic capillary leak syndrome
Source: JCI Insight. 2024 Dec 20;9(24):e173664. doi: 10.1172/jci.insight.173664 (PMC11665552; doi:10.1172/jci.insight.173664)
Supplement: Supplemental data [file jciinsight-9-173664-s226.pdf]

**A**

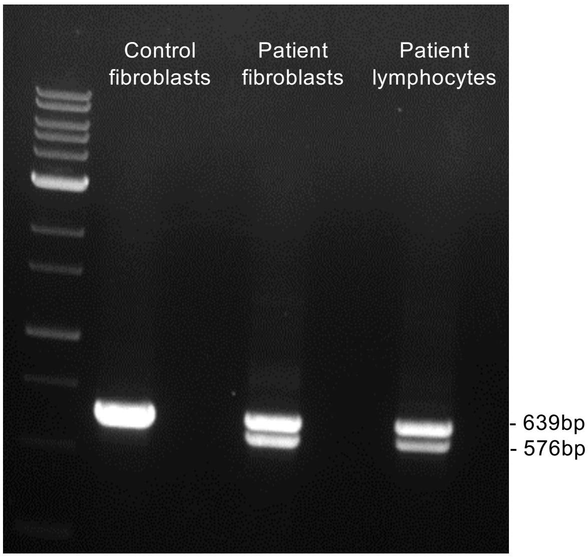


B

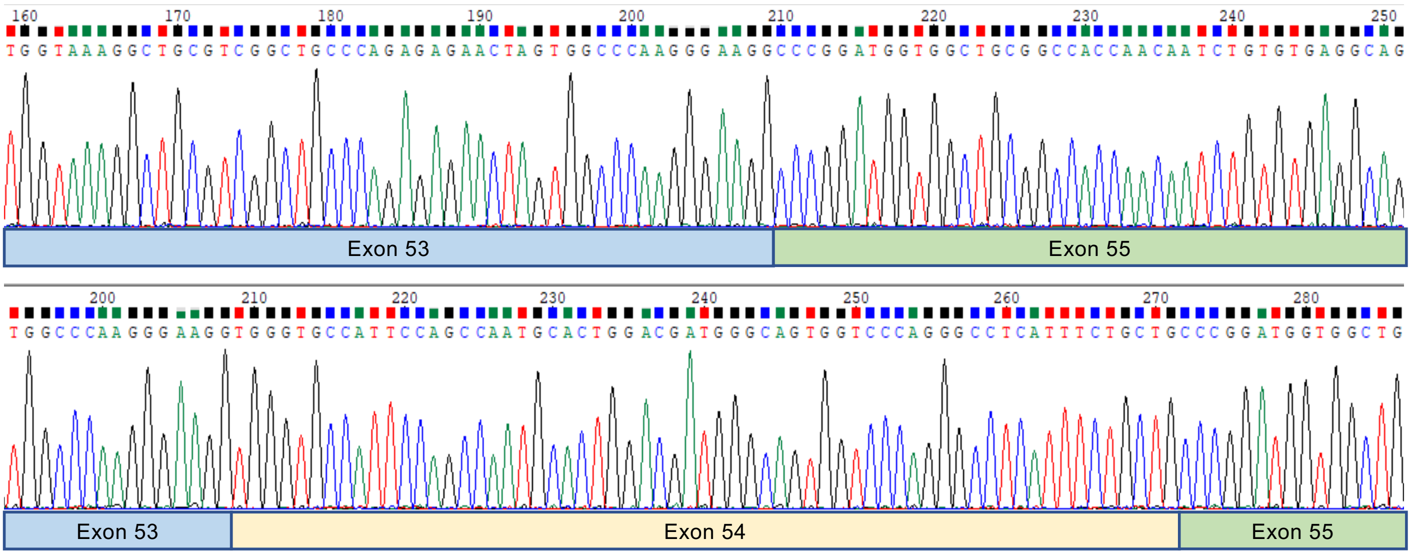


**Supplemental Figure 1**. **Reverse-transcription of SCLS-*TLN1* RNA demonstrates the deletion of exon54 in patient fibroblasts and lymphocytes**.

(**A**) Agarose gel of cDNA from patient and control samples, demonstrating the presence of a lower molecular weight band in patient fibroblasts and lymphocytes (from peripheral blood) as compared to control fibroblasts, indicating mis-splicing. wild-type talin1: 639 bp; SCLS-*TLN1* mutant with skipped exon 54: 576 bp. (**B**) Sanger sequencing at the cDNA level. Upper panel represents the sequence of the SCLS-*TLN1* mutant allele following gel extraction, showing in-frame skipping of exon 54. Lower panel shows the wild-type *TLN1* sequence.

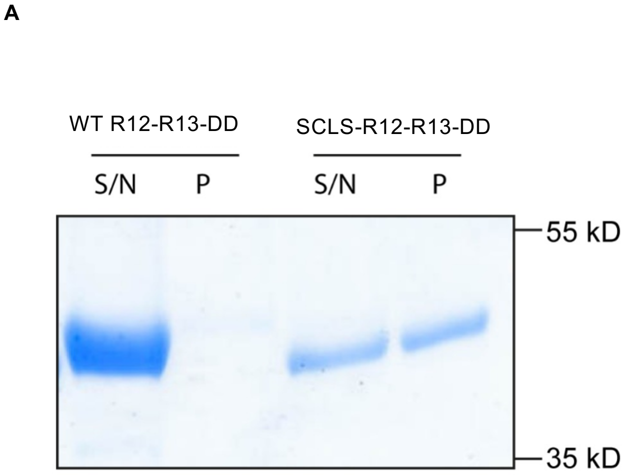


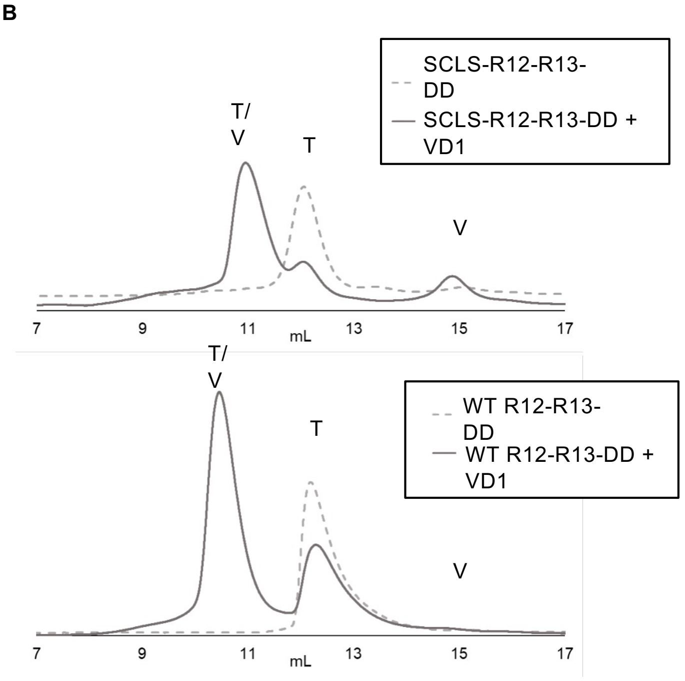


**Supplemental Figure 2**. **Biochemical analysis of the SCLS-R12-DD domain.**

(**A**) Control experiment of the high-speed centrifugation for the actin co-sedimentation experiment. WT R12-R13-DD and SCLS-R12-R13-DD were spun alone at 50,000 x g. WT is seen in the supernatant (S/N) whereas the SCLS-R12-R13-DD was present in the S/N and the pellet (P). (**B**) Size exclusion chromatography (SEC) analysis showing 100 µM SCLS-R12-R13-DD (top) and WT R12-R13-DD (bottom) alone and titrated with 100 µM vinculin domain 1 (VD1). T=talin, V=vinculin, T/V=talin vinculin complex.

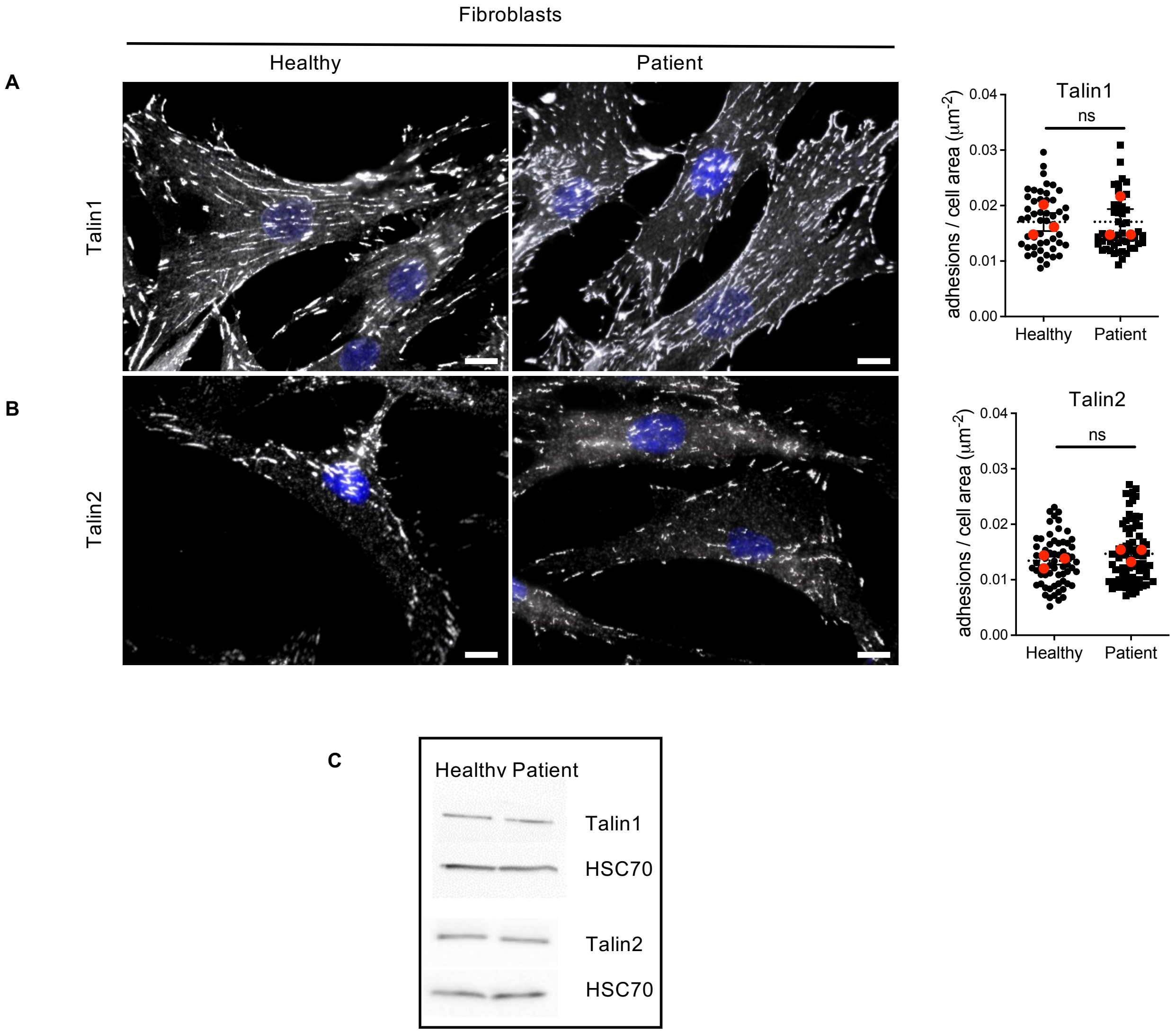


**Supplemental Figure 3. Talin1 and talin2 protein expression and localization is not altered in SCLS patient fibroblasts.**

(**A-B**) Confocal 3D images of healthy donor and SCLS patient fibroblasts with antibodies against talin1 (**A**) and talin2 (**B**). Scale bar 15 μm. Scatter plots display the number of cell-ECM adhesions per cell area ($\mu m^2$) as quantified with IMARIS software, n of cells; talin1: Healthy=50, Patient=50; talin2: Healthy=66, Patient=78. Red dots represent the mean ± SEM of 3 independent experiments. Statistical analysis, 2-tailed unpaired t-test. ns, no statistically significant difference. (**C**) Western blot analysis of the talin1 and talin2 expression in lysates from healthy and patient fibroblast. HSC70 acts as the loading control.

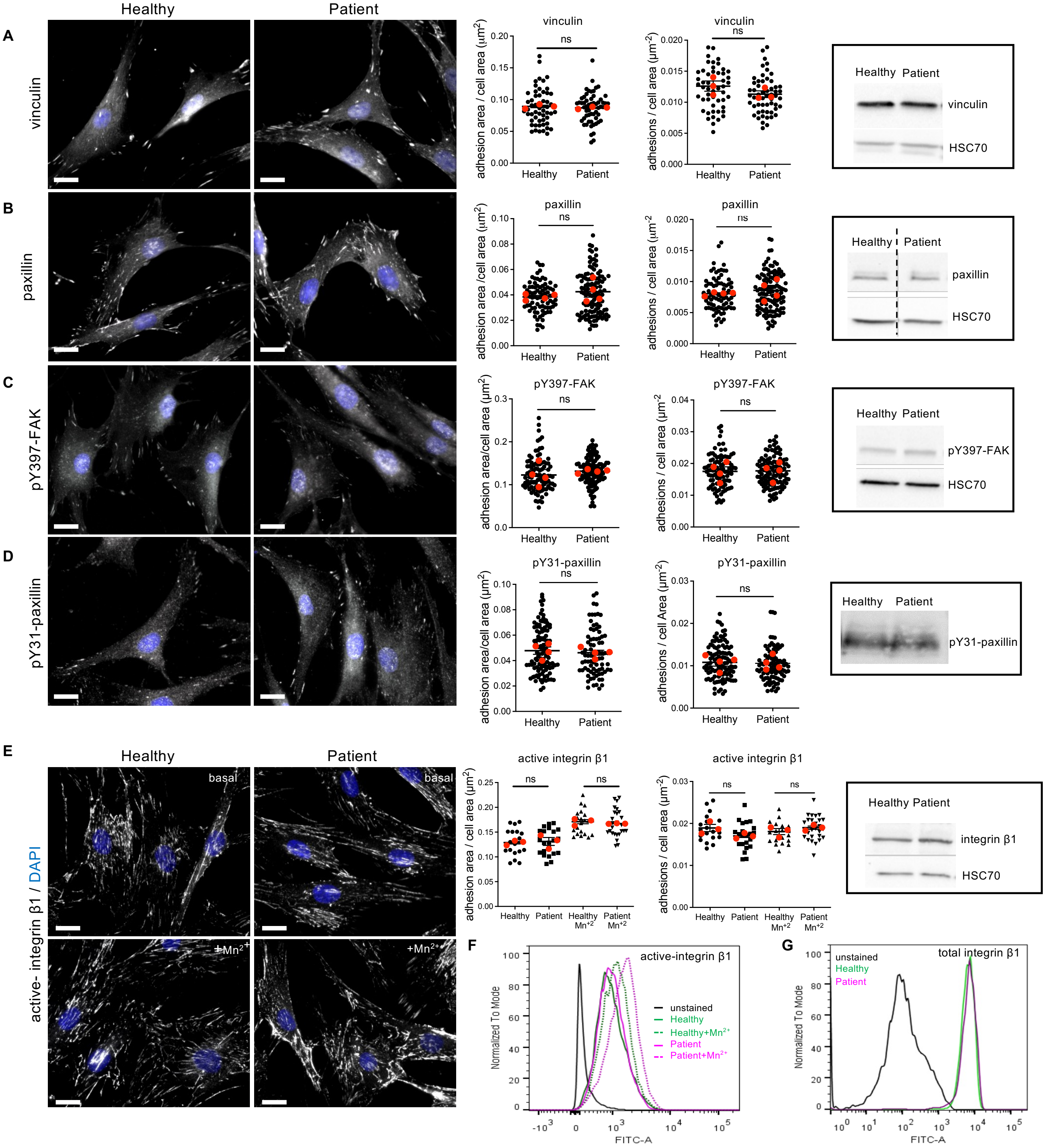

A
B
C
D
E
F
G
Healthy
Patient
vinculin
paxillin
pY397-FAK
pY31-paxillin
active- integrin β1 / DAPI
basal
+Mn2+
adhesion area / cell area (μm2)
adhesions / cell area (μm-2)
ns
HSC70
integrin β1
active integrin β1
Healthy Mn+2
Patient Mn+2
active-integrin β1
total integrin β1
unstained
Healthy+Mn2+
Patient+Mn2+
Normalized To Mode
FITC-A

**Supplemental Figure 4. The SCLS-*TLN1* variant does not affect the adhesion properties of fibroblasts.**

Confocal 3D images of SCLS patient and healthy donor fibroblasts stained for (**A**) vinculin, (**B**) total paxillin, (**C**) pY397-FAK, (**D**) pY31-paxillin and (**E**) active integrin-β1 in the absence (basal) or presence ($Mn^{2+}$) of $MnCl_2$ were used to assess cell-ECM adhesions. Nuclei were stained with DAPI. Scale bars A-D, 15 μm; E, 20 μm. Graphs display the quantification of the cell-ECM adhesion area ($\mu m^2$) and the number of cell-ECM adhesion sites per cell area ($\mu m^2$) measured with IMARIS software. n of cells; vinculin: Healthy=52, Patient=55; total-Paxillin: Healthy=82, Patient=122; pY397-FAK: Healthy=96, Patient=112; pY31-Paxillin: Healthy=110, Patient=81; basal active integrin-β1: Healthy=19, Patient=22; induced active integrin-β1: Healthy=20, Patient=27. Red dots represents the mean ± SEM of 3 independent experiments for A and E and 4 independent experiments for B-D. Statistical analysis, 2-tailed unpaired t-test. ns, no statistically significant difference. Representative western blots of cell-ECM adhesion protein expression in lysates from healthy and patient fibroblasts. Experiments were performed at least twice. HSC-70 acts as the loading control. The lanes for western blot of paxillin (C) were run on the same blot but were noncontiguous. (**F**) Representatives FACS histograms from 3 experiments of patient and control fibroblasts incubated with or without $Mn^{2+}$ and immunostained with the activation epitope-reporting 9EG7 antibody. (**G**) Representatives FACS histograms from 2 experiments of total integrin-β1 cell surface levels in patient and healthy donor fibroblasts.

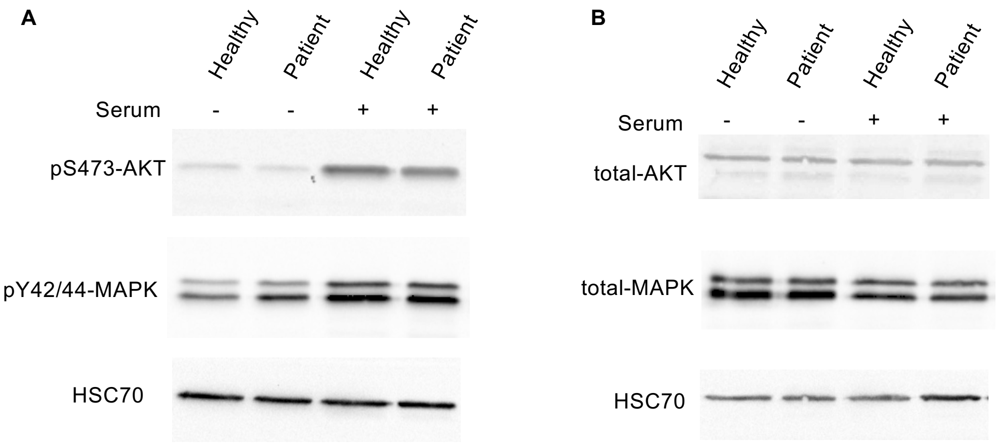


**Supplemental Figure 5. The SCLS-*TLN*1 variant does not affect signal transduction in human fibroblasts.**

Representative western blots of (**A**) pS473-AKT, pY42/44-MAPK and (**B**) total AKT, total MAPK, in lysates of SCLS patient and healthy donor fibroblasts before and after serum stimulation for 10 mins. HSC70 was used as loading control. Experiments were performed three times.

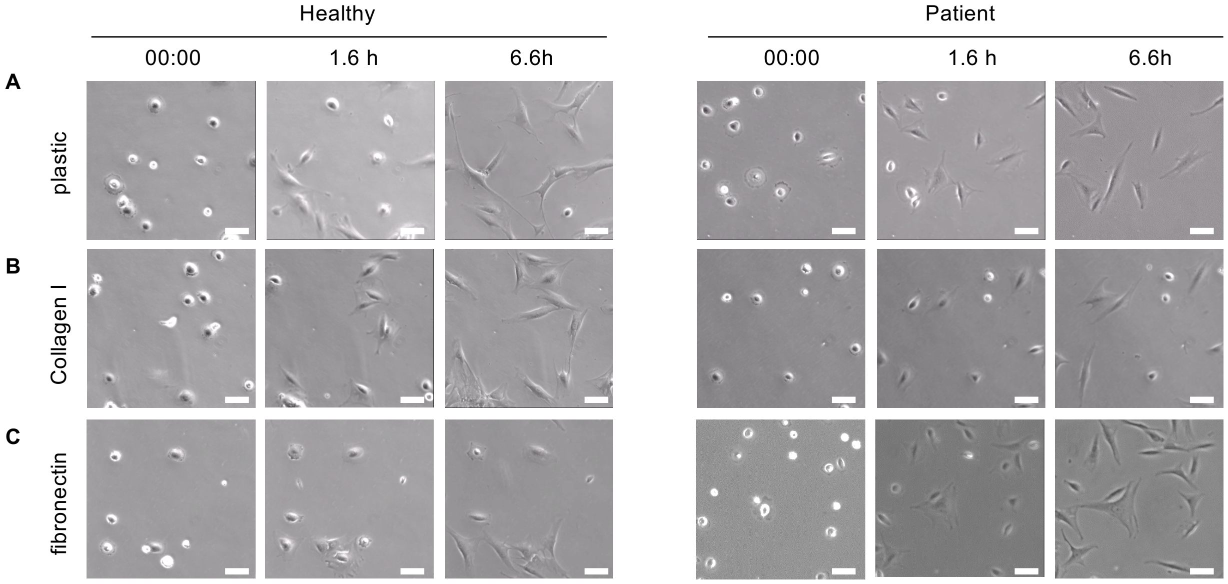


**Supplemental Figure 6**. **Cell adhesion and spreading is unaltered in SCLS patient and healthy fibroblasts.**

Selected frames from different time points of time-lapse imaging showing cell adhesion and spreading on plastic, collagen I and fibronectin of healthy donor and SCLS patient fibroblasts.

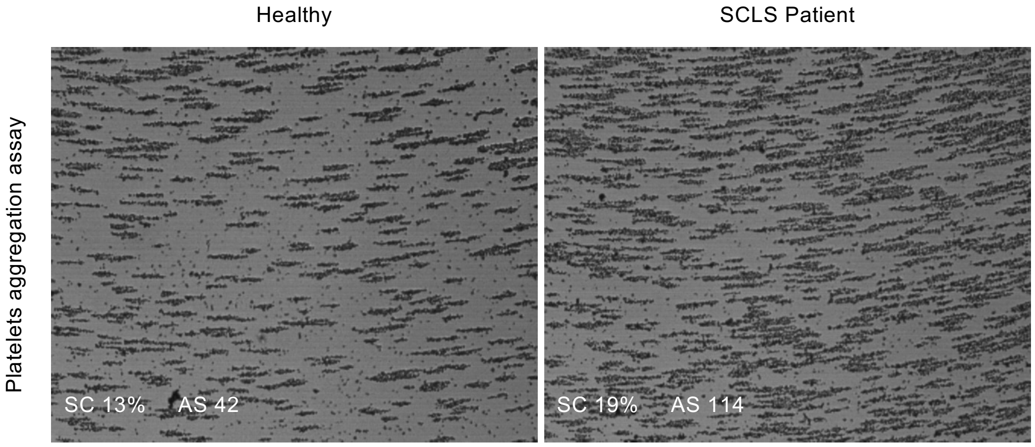


**Supplemental Figure 7**. **Platelets are unaffected in SCLS patient.**

Platelet aggregation assay. Testing the blood sample from both the patient IV-11 and a healthy relative donor, results in aggregate formation on the well surface. SC; surface coverage. AS; average size.

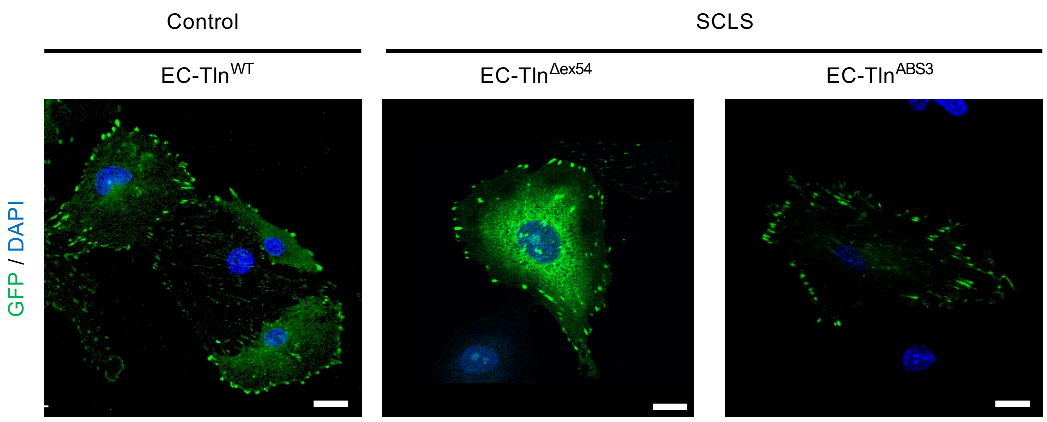


**Supplemental Figure 8. Generation of the SCLS-modeled endothelial cells**.

Representative confocal 3D images of primary mouse talin1 heterozygous ECs transfected with full-length talin1-GFP (EC-Tln$^{WT}$), SCLS-*TLN1* mutant lacking the 21 aa of exon 54-GFP (EC-Tln$^{\Delta ex54}$) or the talin1 ABS3 mutant, R2510A-GFP mutant (EC-Tln$^{ABS3}$) constructs. GFP expression of the constructs (green) demonstrates the localization of talin wild-type and mutant proteins at cell-ECM adhesions. DAPI depicts the nuclei (blue). Scale bar, 20µm.

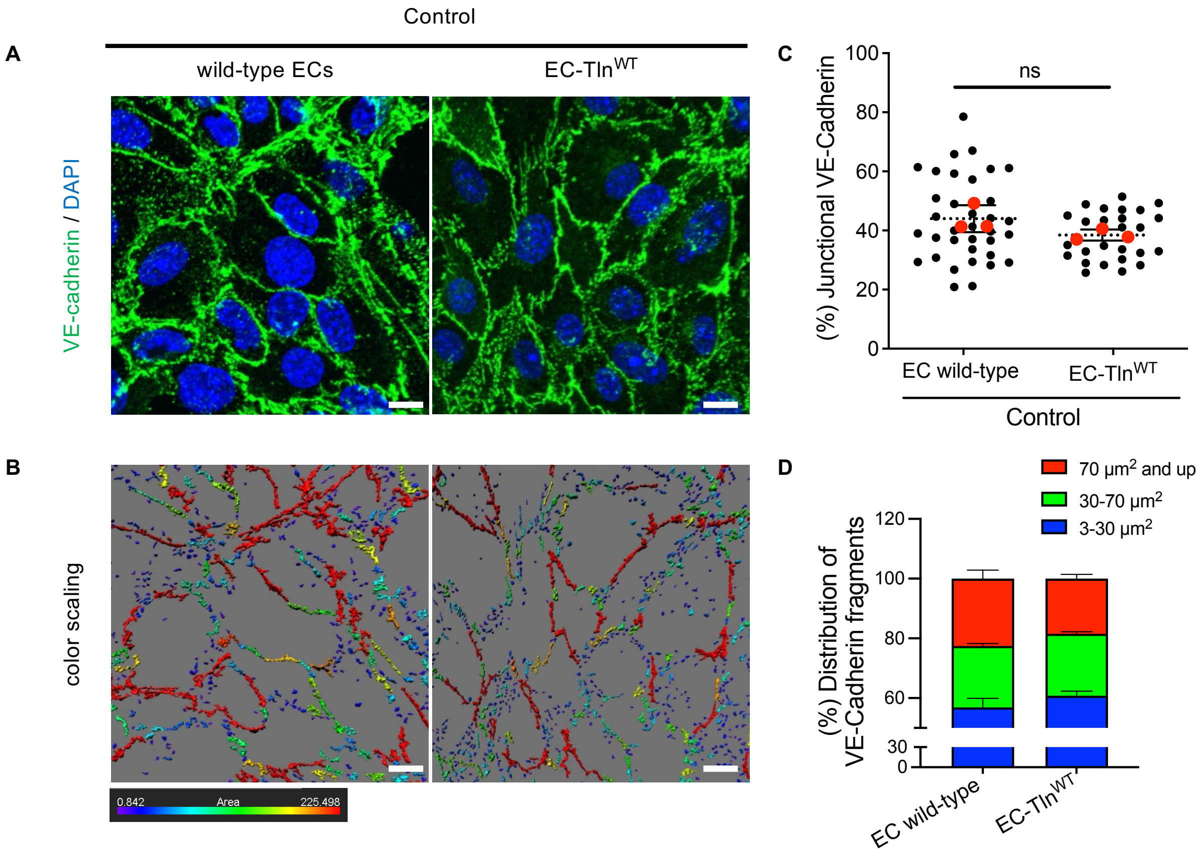


**Supplemental Figure 9. No difference in the morphology of adherens junctions in control endothelial cells.**

(**A**) Representative confocal 3D images of VE-Cadherin (green) immunostained confluent monolayers of mouse primary ECs, either wild-type (WT) or heterozygous for talin1 transfected with full-length talin1 protein (EC-Tln$^{WT}$). Nuclei were stained with DAPI (blue). (**B**) Color scaling of the VE-cadherin staining area, whereby the red color is the highest continuous VE-Cadherin area (>70 μm$^2$), decreasing to smaller areas marked by different colors until it reaches the lowest measurements, which are of blue-violet color (<30 μm$^2$), analyzed by IMARIS. Scale bar, 10 μm. (**C**) Graph displays the quantification of the continuous junctional VE-Cadherin staining area, as represented by the percentage of VE-cadherin staining surfaces above 30 μm$^2$ to the total VE-cadherin staining area. Data represent the mean area per field of monolayer, n fields of view analyzed: wild-type ECs=35; EC-Tln$^{WT}$= 27. Red dots represent the mean ± SEM of 3 independent experiments. Statistical analysis, 2-tailed unpaired t-test. ns, no statistically significant difference. (**D**) Graph displays the distribution of 3 different indexes of VE-cadherin fragments as a percentage of the total VE-cadherin staining surfaces. Data represent the mean ± SEM of 3 independent experiments. n fields of view analyzed, wild-type ECs=30; EC-Tln$^{WT}$= 25.

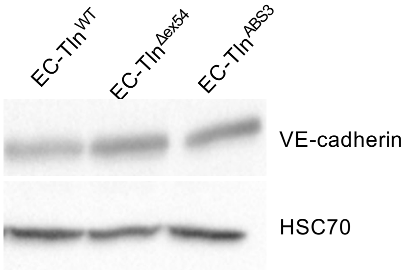


**Supplemental Figure 10. Unchanged VE-cadherin expression in SCLS-*TLN1* mutant and control endothelial cells.**

Representative western blot analysis of mouse primary ECs heterozygous for talin1 expression transfected with full-length talin1 protein (EC-Tln$^{WT}$), the talin1 ABS3 mutation, R2510A (EC-Tln$^{ABS3}$) or the SCLS-*TLN1* mutant lacking 21 aa of exon 54 (EC-Tln$^{\Delta ex54}$). No difference was observed in the total levels of VE-cadherin expression between the control and the mutant-transfected cells. HSC70 acts as loading control. The experiment was performed 3 times.

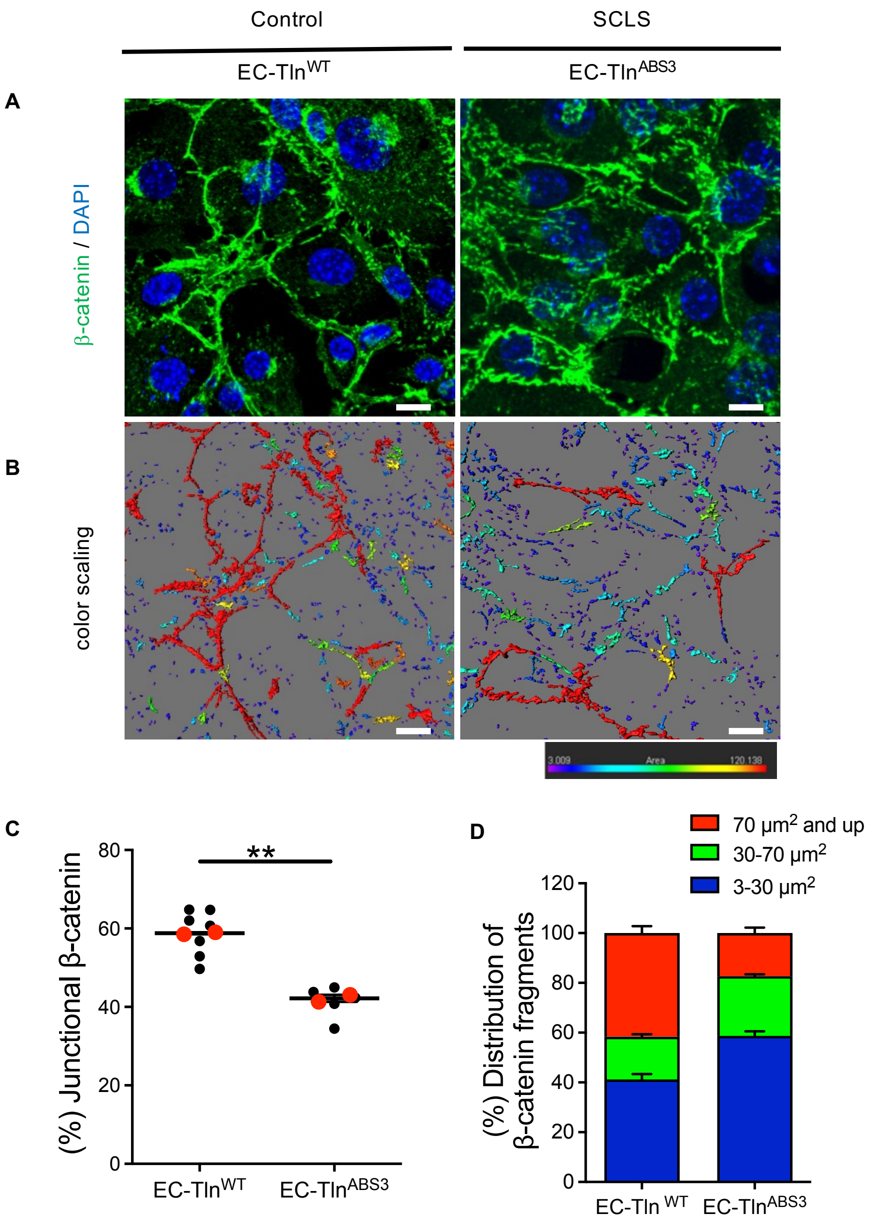


**Supplemental Figure 11. Disrupted adherens junctions visualized using β-catenin staining in talin1-ABS3 mutant endothelial cells.**

(**A**) Representative confocal 3D images of β-catenin (green) stained confluent monolayers of mouse primary ECs heterozygous for talin1 transfected with full-length talin1 protein (EC-Tln$^{WT}$) or the talin1 ABS3 mutation, R2510A (EC-Tln$^{ABS3}$). Nuclei were stained with DAPI (blue). (**B**) Color-scaling of the β-catenin staining area, whereby the red color is the highest continuous β-catenin area (>70 μm$^2$), decreasing to smaller areas marked by different colors until it reaches the lowest measurements, which are of blue-violet color (<30 μm$^2$). Scale bars, 10 μm. (**C**) Graph displays the quantification of the continuous junctional β-catenin staining area, as represented by the percentage of β-catenin staining surfaces above 30 μm$^2$ to the total β-catenin staining area. (**D**) Graph displays the distribution of 3 different indexes of β-catenin fragments as a percentage of the total β-catenin staining surfaces. Red dots represent the mean ±SEM of 2 independent experiments, n fields of view analyzed EC-Tln$^{WT}$=7; EC-Tln$^{ABS3}$= 5. Statistical analysis, 2-tailed unpaired t-test, ** $p<0.005$

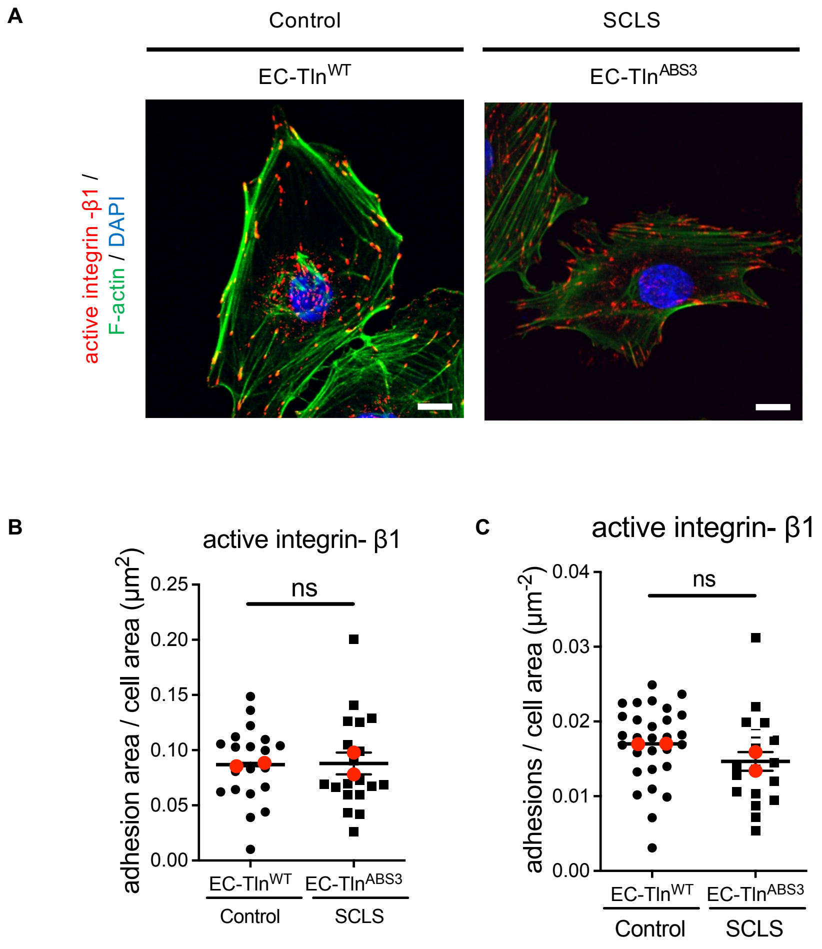


**Supplemental Figure 12. The talin1 ABS3 mutation does not affect integrin activation at cell-ECM adhesions**.

(**A**) Representative confocal 3D images of active integrin-β1 (green) and phalloidin (magenta) stained control EC-Tln$^{WT}$ and talin1 ABS3 mutant, R2510A (EC-Tln$^{ABS3}$) ECs. Nuclei were stained with DAPI (blue). Scale bar, 10µm. (**B-C**) Graphs display the quantification of the cell-ECM adhesion area (µm$^2$) per cell area (**B**) and the number of cell-ECM adhesion sites per cell area (µm$^2$) (**C**) measured with IMARIS software. n of cells; adhesion area/ cell area, EC-Tln$^{WT}$: 20, EC-Tln$^{ABS3}$: 19; adhesions/ cell area, EC-Tln$^{WT}$: 29, EC-Tln$^{ABS3}$: 18. Red dots represent the mean ±SEM of 2 independent experiments. Statistical analysis, 2-tailed unpaired t-test. ns, no statistically significant difference.

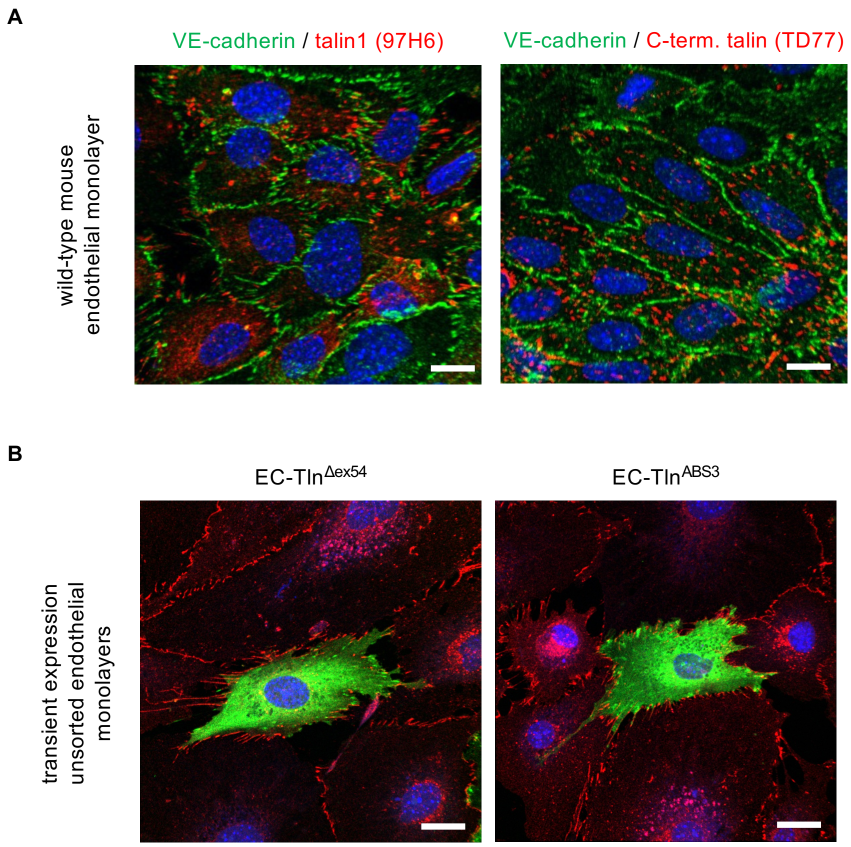


**Supplemental Figure 13. Talin1 is not localized at endothelial adherens junctions**.

(**A**) Representative confocal 3D images of primary wild-type mouse endothelial monolayers immunostained with antibodies against VE-cadherin (green) and talin1 N-terminus (97H6, red) or C-terminus talin (TD77, red). (**B**) Confocal 3D images of VE-cadherin (red) immunostained confluent monolayers of heterozygous talin1 primary ECs transiently transfected with the SCLS-*TLN1* mutant lacking the 21aa of exon 54 (EC-Tln$^{\Delta ex54}$) or the talin1 ABS3 mutation, R2510A (EC-Tln$^{ABS3}$). Nuclei were stained with DAPI (blue). Scale bars, A: 5 µm; B:10 µm.

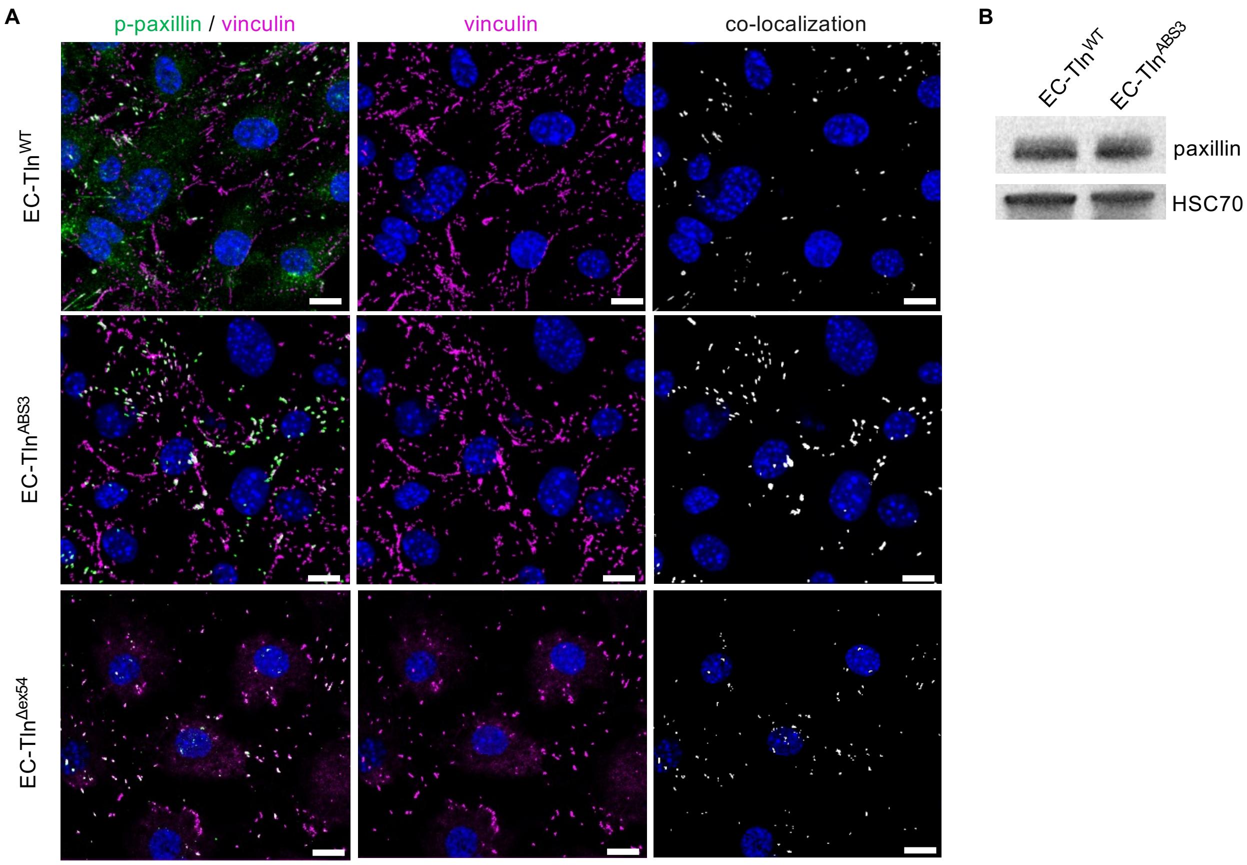


**Supplemental Figure 14. Vinculin is sequestered at cell-ECM adhesion in SCLS-modelled endothelial monolayers.**

(**A**) Representative confocal 3D images of phosphorylated pY31-paxillin (green) and vinculin (magenta) immunostained confluent monolayers of control (EC-$Tln^{wt}$) or the talin1 ABS3 mutant (EC-$Tln^{ABS3}$) or the SCLS-*TLN1* mutant (EC-$Tln^{\Delta ex54}$). Vinculin signal alone (magenta) and the co-localisation signal of vinculin/pY31-paxillin (white) are shown in the middle and right panels, respectively. Nuclei were stained with DAPI (blue). Data represents 2 independent experiments (**B**) Western blot analysis of paxillin expression levels in control and SCLS-modelled ECs. HSC70 acted as a loading control.
