# Supplementary material for: Talin1 dysfunction is genetically linked to systemic capillary leak syndrome
Source: JCI Insight. 2024 Dec 20;9(24):e173664. doi: 10.1172/jci.insight.173664 (PMC11665552; doi:10.1172/jci.insight.173664)

Full unedited blot for figure 7C

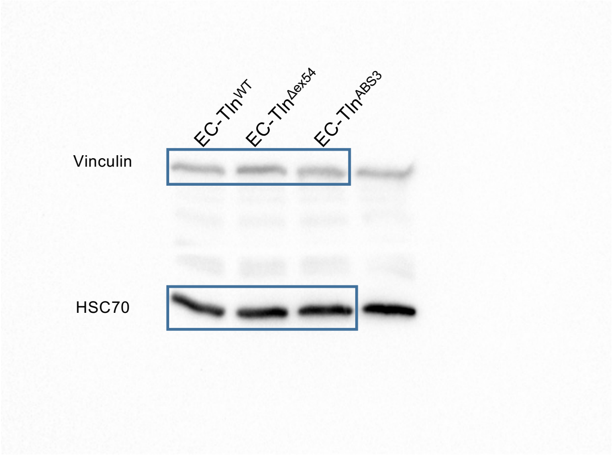

Full unedited blot for supplemental figure 1A

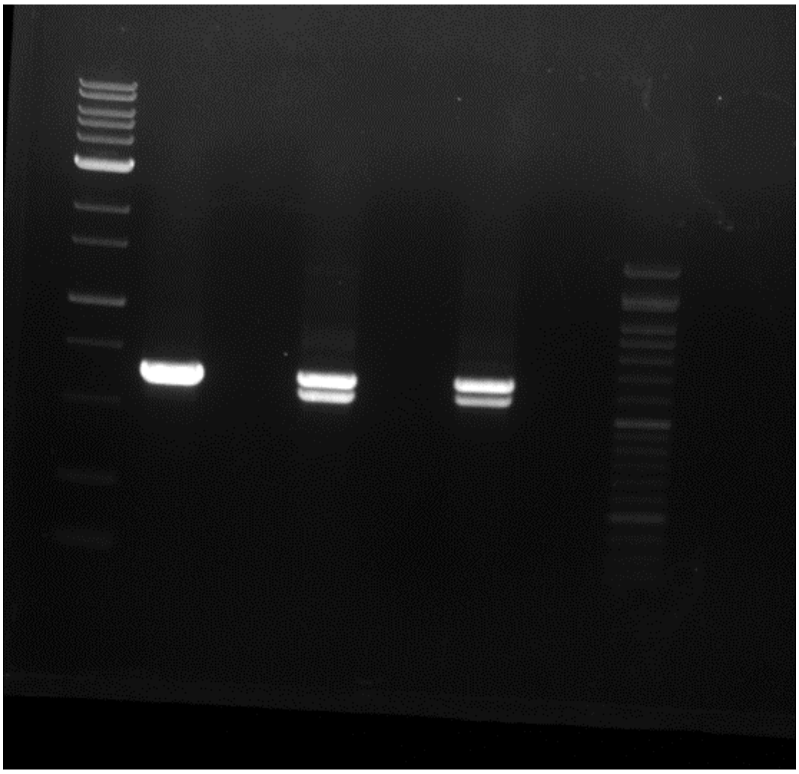

Full unedited blots for supplemental figure 3B

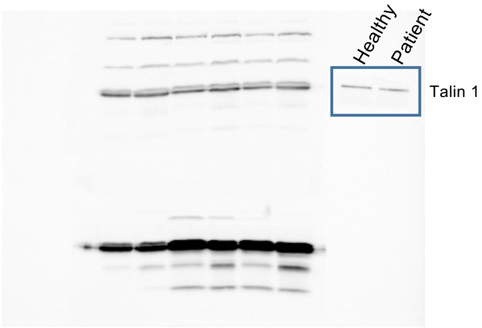

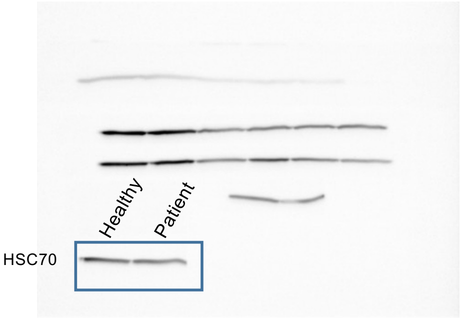

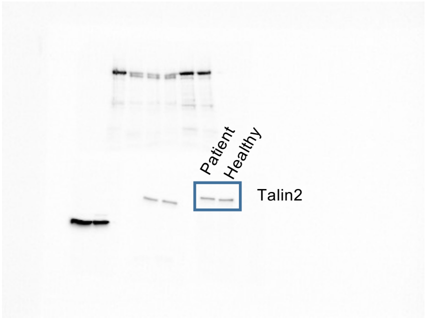

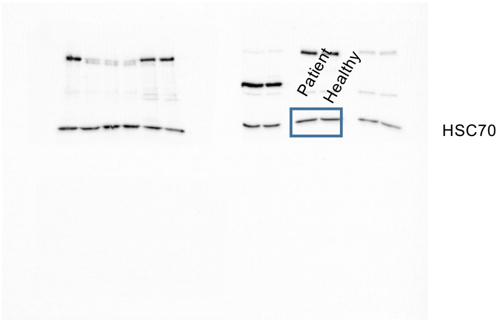

Full unedited blots for supplemental figure 4 A

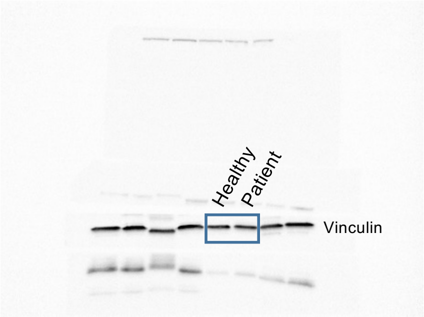

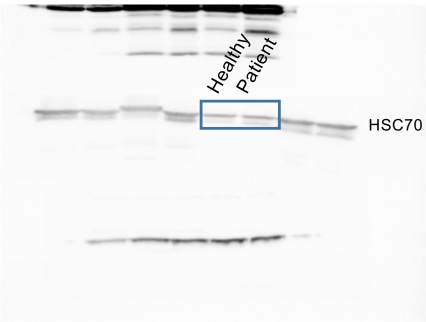

Full unedited blots for supplemental figure 4 B

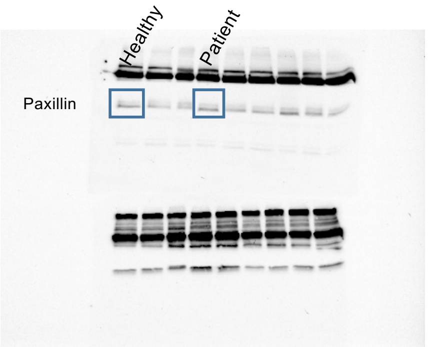

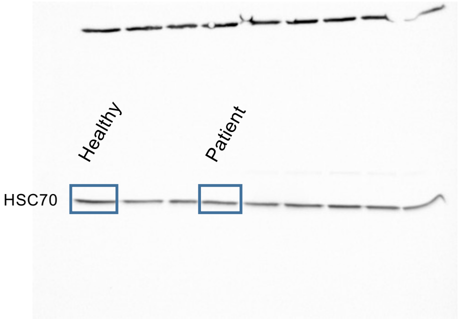

Full unedited blots for supplemental figure 4 C

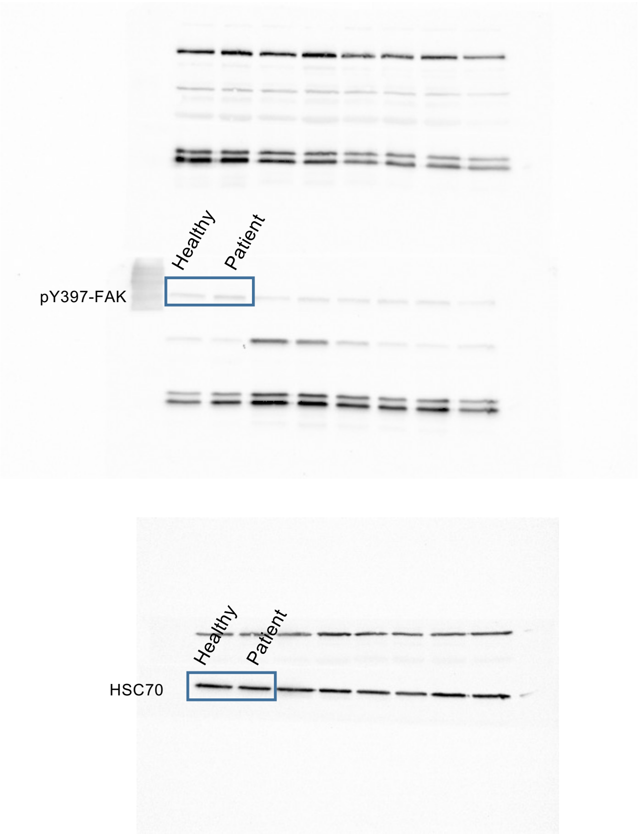

Full unedited blots for supplemental figure 4 D

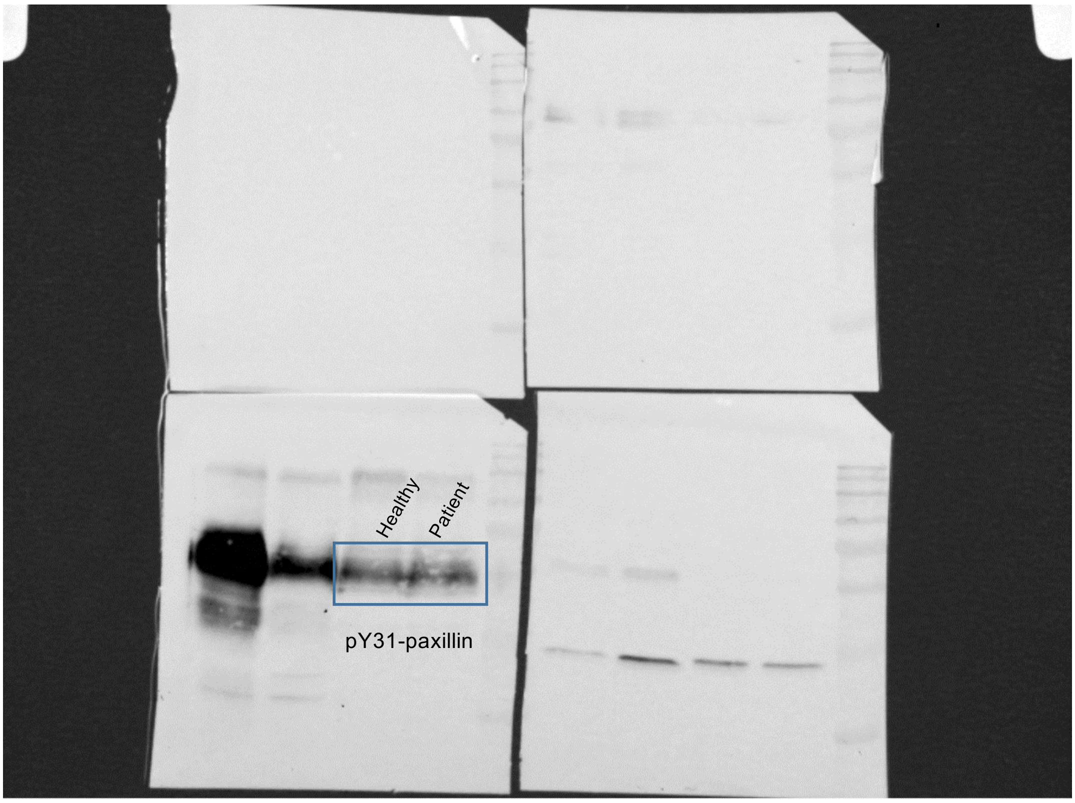

Full unedited blots for supplemental figure 4 E

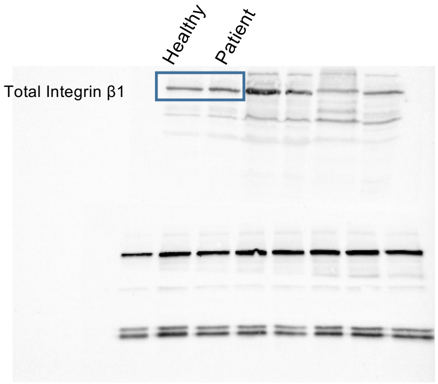

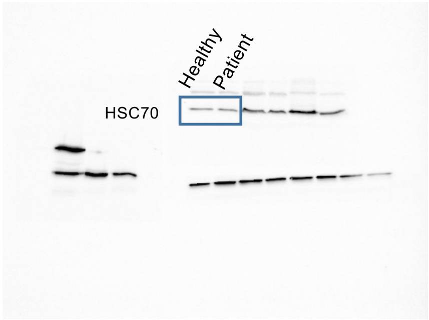

Full unedited blots for supplemental figure 5

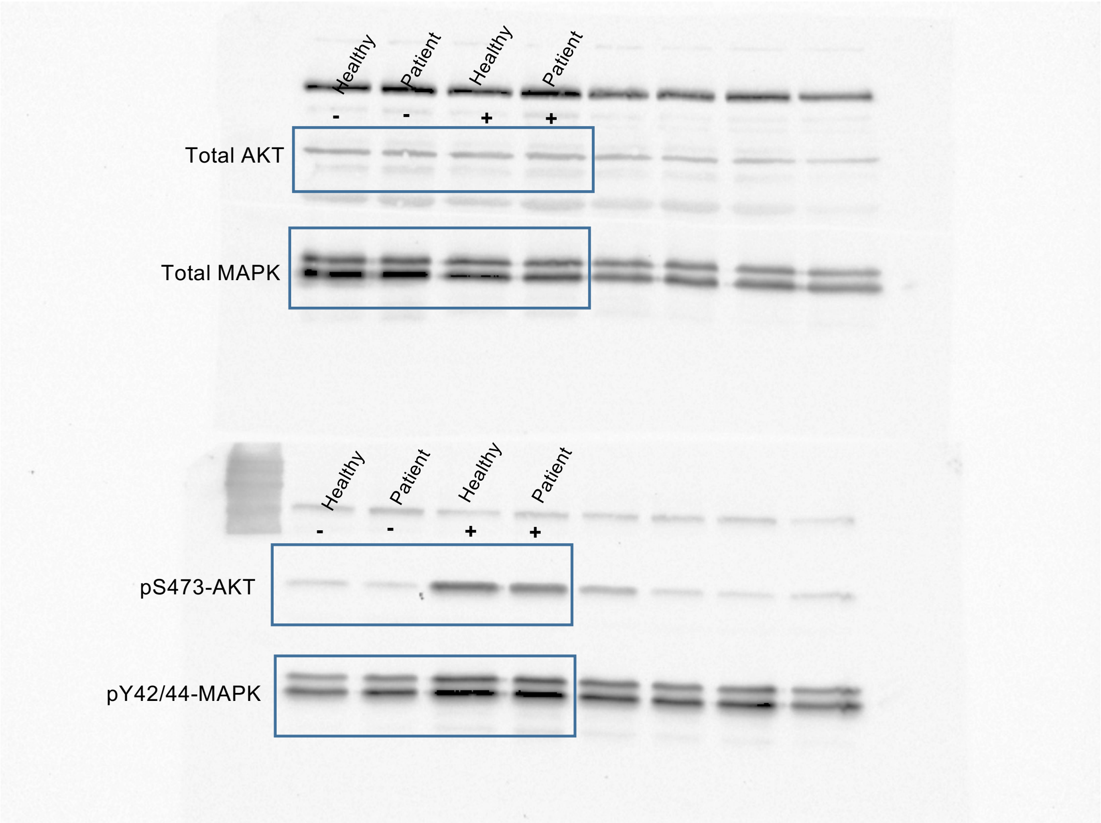

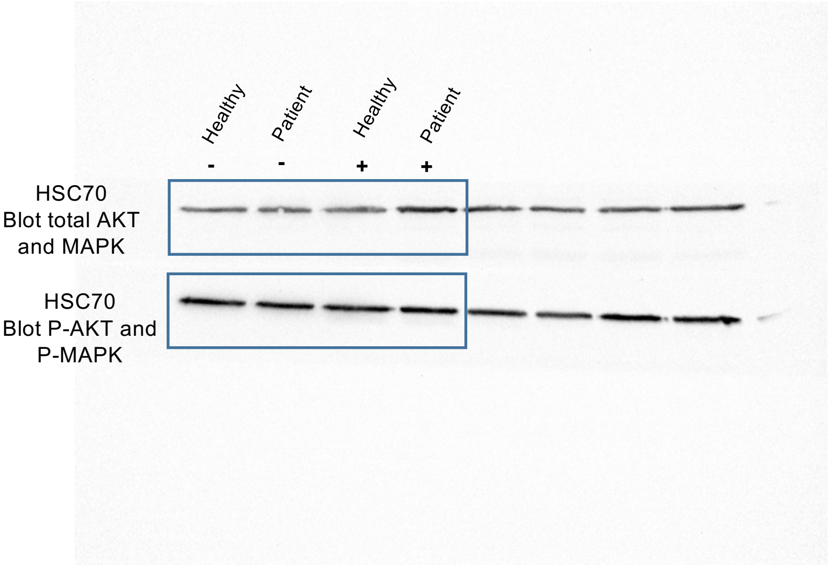

Full unedited blots for supplemental figure 10

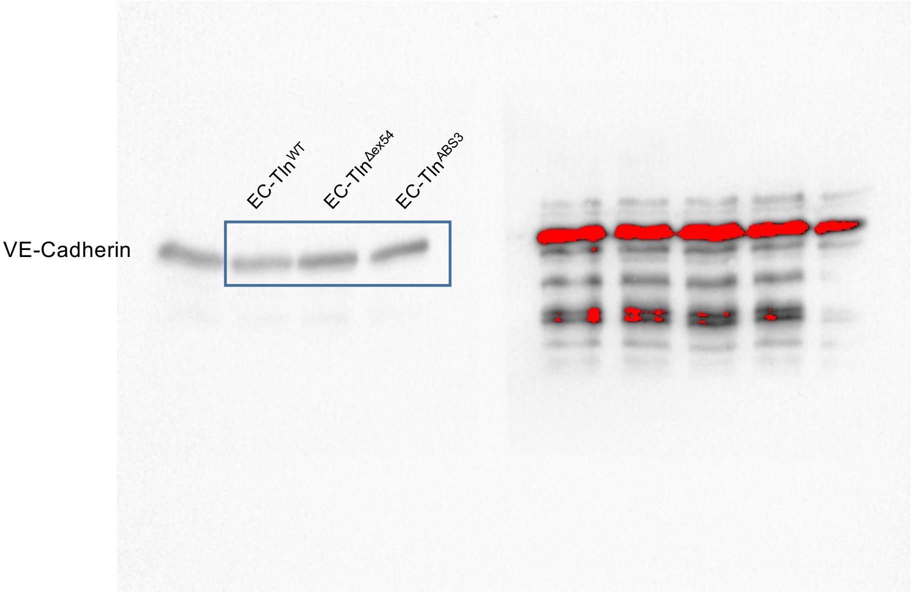

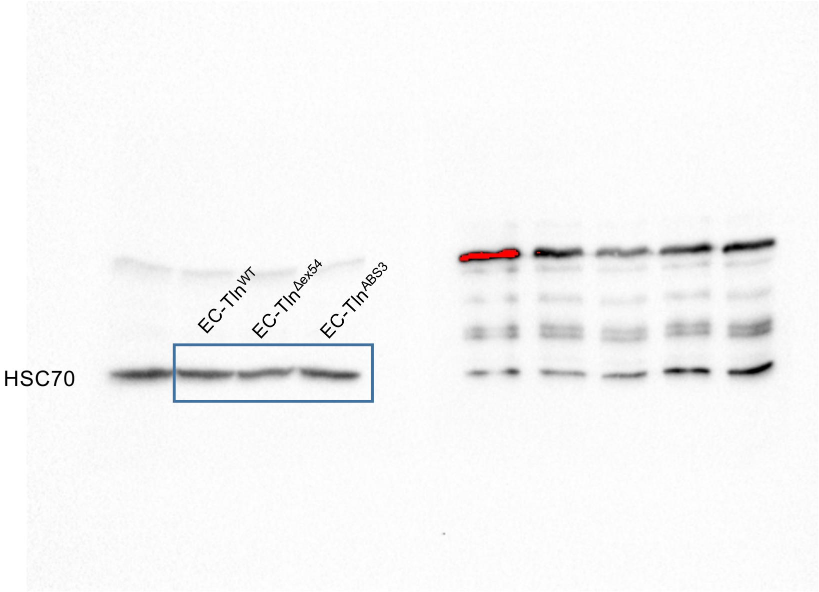

Full unedited blots for supplemental figure 14

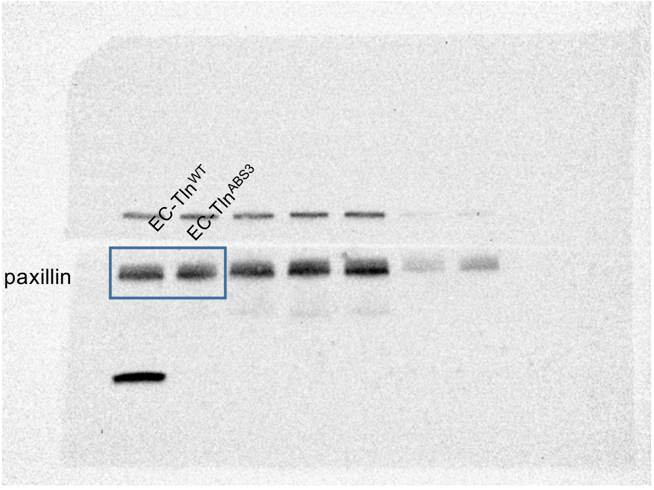

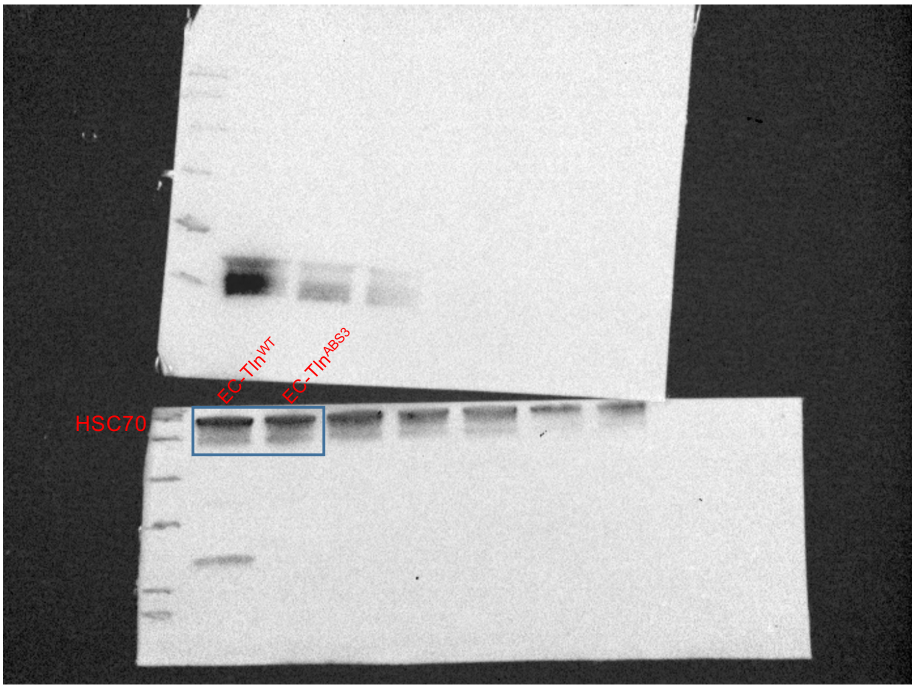

Supplement: Unedited blot and gel images [file jciinsight-9-173664-s227.pdf]
